# Inequalities in Health and Care Among Lesbian, Gay, and Bisexual People Aged 50 and Older in the United Kingdom: A Systematic Review and Meta-analysis of Sources of Individual Participant Data

**DOI:** 10.1093/geronb/gbaa071

**Published:** 2020-05-31

**Authors:** Dylan Kneale, James Thomas, Robert French

**Affiliations:** 1 EPPI-Centre, UCL Institute of Education, University College London, UK; 2 School of Medicine, Cardiff University, UK

**Keywords:** Inequality, Life course, Minority stress, Sexuality, Well-being

## Abstract

**Objectives:**

Modeling the health and care trajectories of lesbian, gay, and bisexual (LGB) is essential to identify inequalities and support needs, yet because of the small sample of LGB people in any one survey, current evidence relies on studies that have poor generalizability and low power. This study assesses the magnitude of health inequalities among older LGB people across 10 outcomes, informed by evidence on the health trajectories and distinct LGB history of the United Kingdom.

**Method:**

A systematic review was conducted of representative data sources on older LGB and heterosexual people’s health and care status in the United Kingdom. Individual Participant Data (IPD) meta-analysis was employed to synthesize data from up to 25 different sources. To account for the intricacies of individual data sets, the analysis employed a two-stage approach where an odds ratio and standard error was calculated for each data set individually, before being meta-analyzed through DerSimonian and Laird random effects models.

**Results:**

Among men aged 50+, being gay, bisexual, or having another nonheterosexual orientation is associated with an increased risk of reporting long-term illness and health-related limitations. Indicators of mental health also suggest that gay and bisexual men are more likely to report low life satisfaction and to have attempted suicide over their life time. Among women, differences are apparent with regards to self-rated health as well as with engagement with risky health behaviors.

**Discussion:**

The findings corroborate the minority stress theory, but they also generate new questions for researchers around when and how these inequalities emerge.

## Sexuality, Equality, and the Health of Older People in the United Kingdom

In many countries older lesbian, gay, and bisexual (LGB) people were born at a time same-sex activity between men was a criminal offence, and where social and legislative conditions permitted discrimination across a wide spectrum of domains for men and women from sexual minorities. In the United Kingdom, even after advances in the 1960s and 1970s such as the decriminalization of same-sex acts between men in 1967 in England and Wales, the legislative and social landscape remained hostile to LGB men and women. During the 1980s, when the last of the baby boom generation were experiencing transitions to adulthood, the onset of the HIV/AIDS epidemic had devastating impact on the health, well-being, and social networks of LGB people. The HIV/AIDS crisis and the government of Margaret Thatcher politicized homosexuality, culminating in the enactment of the controversial Section 28 of the Local Government Act which banned any form of “promotion” of homosexuality including discussion in schools. This galvanized the gay rights movement in the United Kingdom, leading to the establishment of two of the United Kingdom’s best known gay rights movements, Stonewall and OutRage!, in 1989 and 1990, respectively. Since the 1990s, the legislative landscape has become increasingly permissive, so that LGB people can access similar rights and treatment as heterosexual people across a range of domains. Similarly, recent medical advances in antiretrovirals have triggered a drop in new HIV/AIDS infections, and many who were infected now have undetectable levels, effectively halting transmission (Rodger et al., 2019).

Despite these recent advances, the long-term impact of experiences of discrimination on the health and well-being of older LGB people is relatively unknown. In the United Kingdom, discrimination was experienced against a background where LGB movements were muted and largely inaccessible. Between the 1950s and 1980s, LGB movements in the United Kingdom were made up of social elites or protest groups dominated by a few individuals (Kollman & Waites, 2011), unlike federated models in the United States and Europe (Bernstein, 2011; Kollman & Waites, 2011). This also stymied the development of a radical social equality agenda, with direct action in the United Kingdom lagging behind the United States in terms of pride and protest (Downes, 2019). Older LGB people in the United Kingdom experienced greater disconnect from grassroots support structures compared to their U.S. counterparts, compounding feelings of exclusion which may have a direct and long-term impact on health. In contrast, although positive attitudes toward same-sex activity in the United Kingdom and United States were broadly similar (Keleher & Smith, 2012; Park & Rhead, 2013), it was only in 2003 that same-sex activity was legalized across all U.S. states, 36 years after legalization in England and Wales.

In this study we aim to calculate robust estimates of the extent of sexuality-based inequalities in the United Kingdom. Given that the assumptions underpinning a meta-analytic approach, our chosen method in this study, are based on reasonable homogeneity between studies, the inclusion of other contexts including the United States may serve to undermine these assumptions. In this study we focus on UK data alone, and although the exact transferability of estimates of sexuality-based inequalities is unclear, findings from the United Kingdom will have broader salience in illuminating the nature and direction of sexuality-based inequalities.

## Conceptual Underpinnings and Existing Evidence on Sexuality and Health

Recognition that living in a minority status category is deleterious for health has stimulated the development of several theories that provide a framework for exploring sexuality-based health inequalities, the most prominent being minority stress theory (Fabbre, Jen, & Fredriksen-Goldsen, 2018). The theory states that LGB people are at risk of mental health issues from chronic social stressors related to the experience of stigma and prejudice; the concept of social stress has also been extended to physical manifestations of stress. The present study is grounded in examining the long-term impact of exposure social stressors on the health of older LGB people. An earlier systematic scoping review found evidence suggesting that health inequities persist, although the evidence was concentrated around inequality in access to and treatment within social (personal) care settings (Kneale, Henley, Thomas, & French, 2019). The evidence was less conclusive on the extent to which minority stress may manifest in differences in physical and global measures of health among older people. Earlier investigations into the model were suggestive of a link (Meyer, 2003) and later investigations have purposively extended the model to examine the impact of prejudice-related social stress on physical health (Frost, Lehavot, & Meyer, 2015), providing a basis for our current exploration of group-based differences between the physical and mental health status of older LGB and non-LGB people.

Other frameworks have sought to build on the minority stress model to focus more on drivers of health equity. For example, the Health Equity Promotion Model adopts a life course perspective to understand how social position and context, and their intersections, shape both health-promoting and adverse trajectories (Fredriksen-Goldsen et al., 2014). In the context of the present study, this model could support investigation of the mechanisms driving observed inequalities (or health advantages if they exist). However, our focus here is to provide a starting point for such an investigation at a later date through illuminating where health inequalities lie and their magnitude.

## Evidence on Health and Sexuality in Older Age

Existing studies on LGB health in later life predominantly draw on qualitative methods, and the minority of extant quantitative studies often excludes a comparison group of heterosexual people (Almack & King, 2019; Kneale et al., 2019; Westwood et al., 2020). While qualitative studies provide some analytical generalizability to the minority stress hypothesis (Kneale et al., 2019), the extent to which this theory can be generalized across a range of health conditions, and across sociodemographic groups or national settings is less understood. Although minority stress was initially used as a lens to understand inequalities broadly between LGB and heterosexual people, it has increasingly been applied as a lens to explore differences in later life (Detwiler, 2015; Hoy‐Ellis & Fredriksen‐Goldsen, 2017; Wallace, 2018).

In line with reviews of the international literature on health among older LGB people (Addis, Davies, Greene, MacBride-Stewart, & Shepherd, 2009; Brennan, Bauer, Bradley, & Tran, 2017; McParland & Camic, 2016; Potter, Bamford, & Kneale, 2011), UK literature on older LGB people’s health is characterized by a focus on specific issues including HIV/AIDS, sexual health, mental health needs, and substance misuse. Much of this evidence is based on studies that do not make direct comparisons with heterosexual people. Furthermore, evidence that summarizes broad differences in health status, including levels of self-rated health, long-term illness, and limitations due to health or illness, is scarce. This is despite the importance of such broad health indicators in (a) illustrating the impact of different social determinants of health and health inequalities (Bartley & Plewis, 2002); (b) their incorporation into calculations of healthy life expectancy (in the case of self-rated health; Public Health England, 2017) and disability-free life expectancy (in the case of limiting long-term illness; Jagger et al., 2016); as well as (c) being significant antecedents of all-cause mortality (Bentham, Eimermann, Haynes, Lovett, & Brainard, 1995; Idler & Benyamini, 1997; Mossey & Shapiro, 1982).

## Quantitative Evidence on Health and Sexuality and Study Rationale

Quantitative data allowing for the exploration of differences in health status between older LGB and heterosexual people at a population level in the United Kingdom have historically been scarce, and characteristically present methodological drawbacks. Researchers intent on contributing to the evidence base have had to compromise in terms of the generalizability of the sample, for example, in focusing only on those persons with (same sex) cohabiting histories (e.g., Kneale, Sholl, Sherwood, & Faulkner, 2014); or in terms of focusing in on particular populations, for example, clinic attendees (e.g., Bouman et al., 2016). Many quantitative studies using population-level data have identified only small numbers and may be underpowered (Kneale & French, 2018), increasing the risk that significant differences are overlooked. In addition, small sample sizes have compelled researchers to “lump” together diverse intersectional categories across sexual identity, age, and gender.

In response to methodological deficiencies, researchers have occasionally opted to collect new data (e.g., Guasp, 2011), although such a strategy not only represents a high financial cost, but also fails to capitalize on existing UK population-level surveys, which are increasingly collecting data on same LGB people. Individually, these studies are rich in breadth but are compromised by small samples of older LGB people. Evidence synthesis techniques, and particularly meta-analysis, could provide robust evidence through increasing the statistical power of models (Cohen, 1992), compared to relying on individual studies, and reduce the standard error of the (weighted) effect size. Individual Participant Data (IPD) meta-analysis involves the application of meta-analytic methods to participant-level data allowing more flexible statistical analysis (see Riley, Lambert, & Abo-Zaid, 2010). While systematic reviews typically identify papers on a specific topic, in this study we undertook a systematic review of UK data sources that could be used to estimate the magnitude of sexuality-based health inequalities in later life. Our original intention had been to also examine data for transgender people in the United Kingdom, although the low numbers of transgender people and the absence of measures in surveys precluded exploration of transgender health.

## Method

### Scope, Search Strategy, and Data

Data were required to be representative of the United Kingdom or its constituent countries (e.g., Wales or Scotland), or defined regions within the United Kingdom (e.g., South East England), and the main search was confined to the United Kingdom’s largest repository of individual social data hosted by the UK Data Service (UKDS). The UKDS provides access to over 6,000 sources of population, social and economic data including well-known health data (e.g., Scottish and English Health Surveys), and aging data (e.g., the English Longitudinal Study of Ageing [ELSA]). Further details of the search methods are available in the Supplementary Materials. Conduct of the analyses followed the “Preferred Reporting Items for a Systematic Review and Meta-analysis of Individual Participant Data” (Stewart et al., 2015). Confining the search to the UKDS allowed for a systematic approach to the inclusion of data sources in the synthesis, and provided some safeguards around the authenticity, reliability, and logical integrity of included data sources. This could reduce bias arising from including studies that have been inappropriately collected or collated, and bias from including additional data sources haphazardly.

Data sources were included if they: (a) measured sexuality allowing for categorizing people as being LGB or heterosexual; (b) collected data on people aged 50+; (c) collected information on health (defined below); and (d) were collected through probabilistic and representative sampling methods. Data sources that only collected attitudes about LGB people were not eligible and IPD from intervention studies were also ineligible. Outcomes of interest were selected on the basis of findings from an earlier systematic review (Kneale et al., 2019), that highlighted the absence of quantitative estimates of sexuality-based inequalities in broad measures of health status, health behaviors, and mental health, based on comparative population-level estimates:

- **General health measures**: (i) self-rated health, (ii) long-term illness, and (iii) limitations due to health or illness;
- **Measures of health behaviors**: (iv) current smoking (any instance of current smoking—generally the surveys predated e-cigarettes) and (v) heavy drinking (drinking 5 times a week or more);
- **Measures of mental well-being**: (vi) life satisfaction, (vii) suicide attempts, and (viii) suicidal ideation as measures of mental health;
- **Additional health disparities**: (ix) osteoporosis as a measure of health disparity of particular salience to transgender men and women (Sedlak et al., 2017; Wierckx et al., 2012) and (x) a further measure exploring whether LGB older people experienced a greater care “burden” (as carers) was also examined based on previous research (Kneale & French, 2018).

To account for potential differences between LGB and non-LGB people which may confound the observed relationship between sexuality and health, we used a standard set of controls to adjust estimates of sexuality-based differences based on age group, gender (where appropriate in “LGB” models), an indicator of socioeconomic status, retirement status, and marital status. While each study contained a variable reflecting each of these domains, there were differences in measurement between data sets.

### IPD Meta-analysis

All of the data sets used probabilistic sampling necessitating the use of weights to calculate estimates of health inequalities. In some of the data sources, a complex study design had been employed which necessitated accounting for stratified sampling and clustering in the data in order to produce accurate estimates of the odds of experiencing health states in LGB people compared to non-LGB people. To account for the intricacies of individual data sets, the analysis employed a two-stage approach where an odds ratio (OR) and standard error was calculated for each data set individually, before being synthesized in a meta-analytic model (Riley et al., 2010). This approach retained the key advantages of an IPD approach to meta-analysis; for example, greater flexibility in the modeling approach and greater consistency; while allowing the calculation of estimates reflecting the study design from individual studies.

For each study and each outcome measured, a logistic regression model was constructed separately for women, men, as well as a combined male and female model. An unadjusted estimate was obtained as well as an estimate adjusted for the covariates listed above.

As the studies represented UK populations of older LGB people that differed in time and space, random effects meta-analysis models (DerSimonian and Laird method) were initially constructed with the contribution of each study to the pooled effect size reflecting the inverse of each study’s variance as well as the between-study heterogeneity. However, in practice many of the model results constructed with a random effects specification were identical to the results from a fixed effects model due to very low levels of between-study heterogeneity. All data were analyzed using Stata and meta-analysis models constructed through the metan command (Harris et al., 2008). Where moderate heterogeneity was detected (corresponding to an *I*^2^ of 40% or higher; Deeks, Higgins, & Altman, 2011), prespecified subgroup analyses were employed to examine if study-level characteristics explained heterogeneity based on (a) geographic reach of the study; (b) the year of study collection; and (c) the age range of included participants (all were aged 50, although with an upper limit in several studies).

Finally, while the IPD meta-analysis method was intended to overcome issues of estimates being underpowered, we nevertheless faced additional challenges in terms of sparse data within individual data sets (Greenland, Mansournia, & Altman, 2016). As a whole, the health events or states per predictive variable was generally higher than 10 for each model (Vittinghoff & McCulloch, 2007), with the exception of suicide attempts, where the low number of events precluded the use of the data in some cases. However, there was evidence of small categories in some data sets, particularly with respect of our main variable of interest (sexuality); in some data sets fewer than 20 nonheterosexual men and women were identified. Following guidance provided by Greenland et al. (2016), potential sparse data bias was determined where: (a) there were low numbers of LGB people (less than 20 people for a gender specific model); (b) where coefficients were clearly inflated resulting and implausible estimates; and (c) where the magnitude and/or direction of the OR changed dramatically with adjustment of confounders. Where this occurred, an estimate was derived using approximate Bayesian logistic regression using the “penlogit” command in Stata (Discacciati, Orsini, & Greenland, 2015). This had the impact of “shrinking” the log OR toward “zero” through augmenting the data set with data augmentation priors (records that impose the desired priors on the model parameters); these prior values were based on the pooled effect size and the variance based on the next largest study. Due to this procedure being used to adjust estimates from smaller studies, the impact of undertaking this procedure made very little difference to the overall pooled effect size or levels of heterogeneity (confirmed also through sensitivity analyses), although did succeed in adjusting some of the more implausible individual study values. In addition, we also used Firth bias correction where no events were observed in the LGB group, a situation that was only encountered when dealing with rarer events such as suicide attempts (Greenland et al., 2016; Higgins & Green, 2011). Where fewer than 12 LGB men or women were identified in any one data set, the unadjusted ORs were used for both adjusted and unadjusted models for commonly occurring outcomes (including data from the ONS Relationships Module for limitations due to health or illness), although for very rare outcomes the data were not used in models (including certain sweeps of the Scottish Health Survey measuring suicide attempts). Forest plots for each meta-analysis are available in the Supplementary Materials. Full methods for this review were published in an earlier protocol (see Kneale, French, & Thomas, 2018).

## Results

### Review of Data Sets

A total of 1,313 records were retrieved from searches and screened based on title and description. From these, 82 data sets were explored in more detail through downloading questionnaires and checking for eligibility, and from these, a total of 29 different data sets were identified as being eligible. Different outcomes were supported by different numbers of studies, with the largest model (self-rated health) being supported by 25 data sets (see Figure 1 in Supplementary Materials) with just one study (the ELSA) measuring osteoporosis (precluding the ability to meta-analyze the data). Access to the data sets was provided through the standard End User Licence, although access to the largest study, the Integrated Household Survey (Office for National Statistics, 2016), which contained 128,444 people aged 50 and older, was provided through the UK Data Archive’s Secure Lab. A further population-level data set was also considered (South East London Community Health Study) although this was not pursued further because of the very small number of LGB people aged 50+.

### Proportion of People Aged 50+ Identified as LGB

Only one study was discovered allowing for identification of transgender people by measuring gender identity and how this differed from the sex assigned at birth, although the very small number whose gender identity had changed precluded further analysis. The results therefore represent the experiences of LGB people aged 50+. Most included studies asked people about sexual identity and typically respondents could identify as Lesbian, Gay, Bisexual, Heterosexual or as “other” or state “do not know” when asked. These identities were dichotomized, as heterosexual and nonheterosexual (the latter group comprised of all responses except heterosexual). A sizable number of older people also “prefer not to say” when asked about their sexuality (Joloza, Evans, O’Brien, & Potter-Collins, 2010); these responses were not utilized in analyses. Using this approach, the proportion of older people identified as LGB ranged between 0.8% and 5.3% across surveys (1.1%–5.7% among men and 0.7%–5.2% among women, see Table 1). A second way of identifying respondents as LGB in three surveys was to use information on same-sex attraction and same-sex experience. Using a strategy outlined elsewhere (Kneale & French, 2018), we identified respondents as being LGB if they had some, equal, or mainly/exclusively same-sex experience and equal or mainly/exclusively same-sex attraction. Using this method, the proportion of older people identified as LGB ranged between 3.2% and 6.0% across surveys (4.4%–7.8% among men and 2.3%–5.4% among women).

**Table 1.** Dataset Details Including Number and Proportion of People Aged 50 Identified as LGB in Each Survey

	Acronym	Year	Geography	Measurement of sexuality	Age range	Outcomes used										LGB People Aged 50+		LGB Men Aged 50+		LGB Women Aged 50+	
**Dataset and weighted percentages**						Low self- rated health	Long- term illness	limitations due to health/ illness	Low life satisfaction	Suicidal ideation	Suicide attempts	Osteoporosis	Current smoking	Alcohol consumption 5+ days	Care	Weighted proportion	Unweighted number	Weighted proportion	Unweighted number	Weighted proportion	Unweighted number
**Active people Survey** ^**a**^	ActPS	2014	England	I/O	50–100		✓	✓								2.4%	765	2.8%	360	2.0%	405
**Adult Psychiatric Morbidity Survey** ^**b**^	APMS	2007	England	I/O	50–95	✓		✓		✓	✓					5.3%	190	5.4%	90	5.2%	100
**British Social Attitudes Survey** ^**b**^	BSA	2007	Great Britain	I/O	50–98	✓										3.2%	25	5.7%	18	1.2%	<10
**English Longitudinal Study of Ageing** ^**b**^	ELSA	2012	England	E/A	50–98	✓	✓	✓	✓			✓	✓	✓	✓	5.0%	281	5.7%	132	4.3%	149
**Health Education Monitoring Survey** ^**b**^	HEMS	1995 1996 1997 1998	England	I/O	50–55	✓	✓	✓					✓	✓		1.4%	27	1.9%	21	0.7%	<10
**Health Survey for England** ^**b**^	HSE	2010	England	I/O	50–98	✓	✓	✓					✓	✓		1.9%	35	2.9%	23	1.0%	12
		2011	England	I/O	50–100	✓	✓	✓					✓	✓	✓	1.1%	36	1.5%	23	0.7%	13
		2012	England	I/O	50–100	✓	✓	✓					✓	✓	✓	1.3%	42	1.7%	28	0.9%	14
		2013	England	I/O	50–100	✓	✓	✓					✓	✓	✓	1.5%	56	2.0%	34	1.0%	22
		2014	England	I/O	50–100	✓	✓	✓			✓		✓	✓	✓	1.5%	50	1.5%	23	1.5%	27
**National Survey of Sexual Attitudes and Lifestyles** ^**$**^	NATSAL	1990	England	E/A	50–59	✓	✓						✓	✓		3.2%	106	4.4%	63	2.3%	43
		2012	England	E/A	50–74	✓	✓	✓					✓	✓		6.6%	262	7.8%	142	5.4%	120
**Northern Ireland Health Survey** ^**$**^	NIHS	2010–11	Northern Ireland	I/O	50–85+	✓	✓	✓	✓				✓	✓	✓	0.8%	16	1.1%	10	0.6%	<10
		2013–14	Northern Ireland	I/O	50–75+	✓	✓	✓	✓✓				✓	✓	✓	2.6%	29	1.3%	<10	1.4%	21
**ONS Omnibus - Relationships Module** ^**$$**^	ONS Omnib	2004	Great Britain	I/O	50–99			✓								1.8%	10	2.5%	<10	1.3%	<10
**Scottish Health Survey** ^**$**^	SHS	2008	Scotland	I/O	50–98	✓	✓	✓	✓		✓		✓	✓		2.0%	51	2.5%	32	1.6%	19
		2009	Scotland	I/O	50–98	✓	✓	✓	✓		✓		✓	✓		1.8%	61	2.4%	38	1.3%	23
		2010	Scotland	I/O	50–98	✓	✓	✓	✓		✓		✓	✓		3.5%	105	3.7%	50	3.4%	55
		2011	Scotland	I/O	50–98	✓	✓	✓	✓		✓		✓	✓		3.8%	120	4.5%	59	3.2%	61
		2012	Scotland	I/O	50–99	✓	✓	✓	✓				✓	✓		1.5%	35	1.7%	17	1.3%	18
		2013	Scotland	I/O	50–99	✓	✓	✓	✓		✓		✓	✓		1.4%	35	1.7%	16	1.1%	19
**Scottish Attitudes Survey**	SAS	2014^$^	Scotland	I/O	50–99	✓	✓									2.3%	17	2.9%	10	1.4%	<10
		2015^$$^	Scotland	I/O	50–99		✓									2.7%	19	2.8%	10	2.6%	<10
**Understanding Society** ^**$**^	U-SOC	2013	UK	I/O	50–99	✓	✓	✓	✓				✓	✓	✓	2.3%	346	2.8%	182	2.0%	164
**Integrated Household Survey** ^**$**^	IHS	2014	UK	I/O	50–99	✓							✓			1.3%	1106	1.5%	611	1.1%	495

I/O, identity/orientation; E/A, experience/attraction.

^a^Numbers and percentages based on models for LTI.

^b^Numbers and percentages based on models for SRH.

### Inequalities in Self-Rated Health, Long-Term Illness, and Limitations Due to Health or Illness

The results for self-rated health, long-term illness, and health-related limitations showed a sexuality-based inequality, with LGB people aged 50 and older being more likely to report poorer health and illness, although differences were observed by gender. Based on a meta-analysis that included data from 3,031 men and women, the odds of LGB people reporting poorer self-rated health (defined as “not good”) was 1.17 times higher (95% confidence interval [CI]: 1.07–1.28), attenuating slightly to 1.14 in adjusted estimates (95% CI: 1.04–1.25). Gender-stratified models showed the odds of gay and bisexual men reporting not good health were 1.22 times higher in unadjusted models (95% CI: 1.08–1.39), attenuating to 1.12 in adjusted models with the CI including 1.0 (95% CI: 0.99–1.28) providing suggestive, although ultimately inconclusive, evidence of a sexuality-based inequality. In contrast, sexuality-based inequalities were not observed in unadjusted models among women but were evident in adjusted estimates, with LGB women having a higher odds of reporting poorer health (OR: 1.15; CI: 1.01–1.32). Very little between-study heterogeneity was observed, and most model estimates were identical to those obtained from a fixed effects model (Table 2).

**Table 2.** Meta-analysis Results for the Odds of Self-Rated Health, Long-Term Illness and Health-Related Limitations Among People Aged 50 and Older by Sexual Orientation

					Unadjusted estimates				Adjusted estimates			
	Model	Studies (*N*)	LGB people (*N*)	Heterosexual people (*N*)	Odds ratio	Confidence interval	*I* ^2^	τ ^2^	Odds ratio	Confidence interval	*I* ^2^	τ ^2^
Self-rated health	Overall	25	3,031	159,371	1.171	1.069–1.282	0.0%	0.000	1.142	1.042–1.252	0.0%	0.000
	Male	25	1,630	73,016	1.223	1.080–1.386	0.0%	0.000	1.124	0.991–1.276	0.0%	0.000
	Female	25	1,401	86,355	1.086	0.890–1.327	36.9%	0.066	1.153	1.010–1.317	0.0%	0.000
Long-term illness	Overall	24	2,495	90,730	1.037	0.886–1.214	49.0%	0.054	1.009	0.855–1.191	51.6%	0.063
	Male	24	1,282	39,685	1.239	1.078–1.423	0.0%	0.000	1.178	1.022–1.358	0.0%	0.00
	Female	24	1,213	51,045	0.920	0.730–1.159	51.0%	0.112	0.924	0.738–1.159	47.0%	0.102
Limitations due to health or illness	Overall	23	2,521	89,188	1.125	0.978–1.293	36.8%	0.031	1.092	0.940–1.268	42.4%	0.048
	Male	23	1,277	39,047	1.289	1.123–1.481	0.0%	0.000	1.202	1.045–1.382	0.0%	0.000
	Female	23	1,241	50,141	1.074	0.922–1.249	8.6%	0.010	1.055	0.912–1.220	5.0%	0.006

*Note.* LGB = lesbian, gay, and bisexual.

Among women, there was no conclusive evidence of a difference in the odds of reporting a long-term illness by sexuality (adjusted OR: 0.92; 95% CI: 0.73–1.16) despite the model including data from 1,213 female participants. In contrast, gay and bisexual men were found to have an elevated odds of experiencing long-term illness (adjusted OR: 1.18; 95% CI: 1.02–1.36) based on a model that included reports from 1,282 gay and bisexual men. Little heterogeneity was observed in the model for men, although greater levels of heterogeneity were observed in the model for women. In the model for LGB women study-level factors (geographic reach, year of study collection, and age range of participants) did not yield an explanation for high heterogeneity.

An almost identical set of results to those for long-term illness was obtained when exploring sexuality-based inequalities in *limitations* due to illness. Gay and bisexual men were substantially more likely to report living with a health-related limitations (adjusted OR: 1.20; 95% CI: 1.05–1.32), an effect that was relatively homogenous across studies based on the very low levels of heterogeneity. Meanwhile, the results for women were inconclusive and suggestive of very little increased risk of LGB women reporting health-related limitations, based on models that included over 1,200 women. Unlike the results above for long-term illness, the results for health-related limitations suggested that the effects across studies were relatively uniform in suggesting that being LGB neither raised nor lowered the risk of limiting illness among women.

### Inequalities in Suicide Attempts, Suicidal Ideation, and Life Satisfaction

Inequalities in the mental health of LGB men and women aged 50 and older were apparent across different indicators of mental health, and particularly among men (Table 3). Inequalities were particularly striking in the raised risk of gay and bisexual men reporting attempts of suicide across their life course, with the odds being over twice as high compared to heterosexual men (adjusted OR: 2.29; 95% CI: 1.19–4.42). Despite the meta-analysis including reports from four studies, the number of gay and bisexual men included in the model remained relatively low (124), and it was not possible to explore the high heterogeneity sufficiently. The results from a model containing one study also suggested that gay and bisexual men aged 50+ were twice as likely to report thoughts of taking their own lives (adjusted OR: 2.03; 95% CI: 1.04–3.94).

**Table 3.** Meta-analysis Results for the Odds of Suicide Attempts, Suicidal Ideation, and Low Life Satisfaction Among People Aged 50 and Older by Sexual Orientation

					Unadjusted				Adjusted			
	Model	Studies (*N*)	LGB people (*N*)	Heterosexual people (*N*)	Odds ratio	Confidence interval	*I* ^2^	τ ^2^	Odds ratio	Confidence interval	*I* ^2^	τ ^2^
Suicide attempts	Overall	4^a,b,c^	258	6,988	2.250	1.184–4.277	20.9%	0.106	2.034	1.169–3.538	11.3%	0.042
	Male	4^a,b,c^	124	3,109	3.418	1.708–6.840	0.0%	0.000	2.290	1.187–4.420	0.0%	0.000
	Female	4^a,b,c^	134	3,879	0.936	0.794–4.717	14.2%	0.149	1.881	0.969–3.651	0.0%	0.000
Suicidal ideation	Overall	1	189	3,532	1.445	0.919–2.274	—	—	1.310	0.825–2.079	—	—
	Male	1	90	1,519	2.523	1.304–4.884	—	—	2.026	1.041–3.943	—	—
	Female	1	99	2,013	0.846	0.415–1.727	—	—	0.815	0.393–1.690	—	—
Low life satisfaction	Overall	10	1,044	38,136	1.439	1.173–1.766	0.0%	0.000	1.260	1.028–1.544	0.0%	0.000
	Male	10	525	17,050	1.630	1.230–2.162	0.0%	0.000	1.329	1.006–1.756	0.0%	0.000
	Female	10	519	21,086	1.325	0.999–1.757	0.0%	0.000	1.239	0.943–1.628	0.0%	0.000

*Note.* LGB = lesbian, gay, and bisexual; LGBT = lesbian, gay, bisexual, and transgender.

^a^Scottish Health Survey 2009 collected data on suicide attempts only from a subset of survey respondents and data are not included in the analysis due to very low number of LGBT respondents included in the subset which could not be disaggregated by gender.

^b^Scottish Health Survey 2013 collected data on suicide attempts only from a subset of survey respondents and not included in the analysis due to very low number of LGBT respondents included in the subset.

^c^Scottish Health Survey 2010 collected data on suicide attempts only from a subset of survey respondents and not included in the analysis due to very low number of LGBT respondents included in the subset.

A model including 525 gay and bisexual older men indicated that sexuality-based mental health inequalities also encompassed reports of lower life satisfaction (adjusted OR: 1.26; 95% CI: 1.01–1.76). Sexuality-based inequalities were not observed as consistently among women. LGB women aged 50+ were somewhat more likely to report suicide attempts (based on reports from 134 women) and low life satisfaction (based on 519 women) although this evidence was inconclusive. The model for life satisfaction among women showed low levels of statistical heterogeneity, although visual inspections of the data did suggest that differences were apparent, and there were some differences in the phrasing of the question across studies (see Supplementary Materials).

### Inequalities in Osteoporosis and the Provision of Care

Only one study provided information on the risks of osteoporosis by sexuality, precluding meta-analysis. The results suggested that older LGB women were at elevated risk, although the model was underpowered.

Meta-analyses of the provision of care suggested that neither LGB men nor women were more likely than heterosexual people to provide care to a family member or loved one. However, there were high levels of heterogeneity visible in both models, with effect sizes for men and women varying both in terms of magnitude and direction. Generally, investigations into heterogeneity were uninformative, although for men we found that the risk of sexuality-based differences in care provision was patterned by the country in which the study took place. Among six studies conducted in England, gay and bisexual men were more likely to report being a carer (adjusted model: OR: 1.38; 95% CI: 1.02–1.86), with no heterogeneity detected in the subgroup; studies conducted in other countries provided inconsistent evidence (Table 4).

**Table 4.** Meta-analysis Results for the Odds of Care Provision and Osteoporosis Among People Aged 50 and Older by Sexual Orientation

					Unadjusted				Adjusted			
	Model	Studies (*N*)	LGB people (*N*)	Heterosexual people (*N*)	Odds ratio	Confidence interval	*I* ^2^	τ ^2^	Odds ratio	Confidence interval	*I* ^2^	τ ^2^
Provision of care to another	Overall	15	1,347	53,909	1.004	0.818–1.232	37.5%	0.052	1.061	0.874–1.288	29.3%	0.039
	Male	15	673	24,395	1.120	0.811–1.547	46.5%	0.155	1.224	0.918–1.631	36.0%	0.104
	Female	15	674	29,514	0.921	0.694–1.223	34.0%	0.092	0.900	0.676–1.200	39.9%	0.113
Osteoporosis	Overall	1	282	3,617	1.136	0.699–1.845	—	—	1.352	0.834–2.191	—	—
	Male	1	133	2,530	0.514	0.153–1.728	—	—	0.467	0.130–1.678	—	—
	Female	1	149	3,087	1.496	0.882–2.539	—	—	1.638	0.963–2.789	—	—

*Note.* LGB = lesbian, gay, and bisexual.

### Inequalities in Smoking and Alcohol Consumption

Models for both high frequency of alcohol consumption and current smoking were supported by a large number of studies (22 and 23 studies, respectively), with data from over 800 nonheterosexual men and women, respectively, included in the models (Table 5). LGB women were substantially more likely to smoke than heterosexual women (adjusted OR: 1.23; 95% CI: 1.05–1.44) with negligible heterogeneity detected, indicating a broadly consistent pattern. There were also indications that LGB women were more likely to drink frequently (typically on five or more days a week, see Supplementary Materials) than heterosexual women (adjusted OR: 1.31; 95% CI: 0.95–1.79), although the CI for this model included 1, indicating that the evidence was inconclusive. No consistent evidence was found for men having higher or lower risks of smoking or high-frequency drinking, and although high levels of between-study heterogeneity were detected, planned explorations were not fruitful in identifying study-level drivers of heterogeneity.

**Table 5.** Meta-analysis Results for the Odds of High-Frequency Drinking and Smoking Among People Aged 50 and Older by Sexual Orientation

					Unadjusted				Adjusted			
	Model	Studies (*N*)	LGB people (*N*)	Hetero-sexual people (*N*)	Odds ratio	Confidence interval	*I* ^2^	τ ^2^	Odds ratio	Confidence interval	*I* ^2^	τ ^2^
Currently smoking	Overall	23	2,420	114,291	1.259	1.110–1.428	9.8%	0.007	1.080	0.945–1.234	12.6%	0.011
	Male	23	1,314	55,800	1.114	0.855–1.452	53.1%	0.571	0.915	0.718–1.168	43.8%	0.011
	Female	23	816	33,087	1.321	1.121–1.556	1.6%	0.002	1.229	1.047–1.442	0.0%	0.000
High-frequency alcohol consumption	Overall	22	1,797	64,463	1.209	1.035–1.412	24.4%	0.026	1.191	1.029–1.379	16.2%	0.016
	Male	22	950	28,972	1.085	0.910–1.294	10.6%	0.016	1.127	0.932–1.366	20.5%	0.034
	Female	22	847	35,491	1.328	0.907–1.946	62.3%	0.367	1.271	0.911–1.773	54.3%	0.253

*Note.* LGB = lesbian, gay, and bisexual.

## Summary and Discussion

### Summary

Among men aged 50 and older, being gay, bisexual, or having another nonheterosexual orientation is associated with an increased risk of reporting a long-term illness and limitations due to health or illness. Gay and bisexual men are more likely to report low life satisfaction and to have attempted suicide over their life time. In these analyses, we were unable to examine the extent to which the elevated levels of long-term illness overlapped with poorer mental health, although the relationship between poorer mental health and poorer physical health has been theorized and documented both in the general literature (Firth et al., 2019), as well as in literature focused on LGB people (Fredriksen-Goldsen et al., 2013; Frost et al., 2015). Similarly, we were unable to examine the extent to which higher levels of long-term illness overlapped with instances of HIV/AIDS. Ageing with HIV/AIDS is increasingly common among older gay and bisexual men (Owen & Catalan, 2012) although most of the data included here were collected before widespread use of effective antiretrovirals (Rodger et al., 2019). If living with HIV provides an explanation for the higher levels of long-term illness observed in the current data, then new therapies may change the significance of this finding in the near future.

We find that among women, differences in health are apparent with regards to self-rated health as well as with engagement with risky health behaviors. There are clear connections between these, with the higher levels of smoking and frequent alcohol consumption a possible contributor to poorer self-rated health. The odds of LGB women reporting “not good” health are 1.15 times higher than heterosexual women and while this differential is relatively modest, this may have substantial impacts on a population level. The odds of LGB women being current smokers was substantially higher, and there were also indications that LGB women were more likely to drink frequently, although this latter result was not significant and there was substantial heterogeneity. Nevertheless, the results provide indications LGB women may have riskier physical health behaviors, with a similar sexuality-based inequality not observed among men in health behaviors. Certainly, the meta-analyses provide suggestive evidence that smoking cessation interventions may fail to reach sexual minority women. Previous systematic reviews have also indicated an absence of specific interventions for LGB women (Rizer, Mauery, Haynes, Couser, & Gruman, 2015). The reason for the emergence of sexuality-based differences for women and not men in health behaviors is unclear. One factor may be that social spaces for gay and bisexual men have historically been a focal point for public health interventions in a way that social spaces for nonheterosexual women have not (Leibel, Lee, Goldstein, & Ranney, 2011).

### Limitations

While the overall findings of this study are relevant to a broad readership in illuminating the nature and overall magnitude of sexuality-based inequalities, the precise estimates are limited by the UK focus. Incorporation of international data, for example, from the United States, was considered although was ultimately discounted because of contextual differences that could undermine the underpinning assumptions of reasonable homogeneity in IPD meta-analysis. Furthermore, differences within countries such as the United States, where legislation reflected a patchwork of rights and sanctions on same-sex activity (Bernstein, 2011), could become difficult to reconcile and interpret in cross-country comparisons. The focus on the United Kingdom also allowed us to remain systematic about the identification of data sets to incorporate in the synthesis, helping to avoid bias. However, incorporating U.S. and other data may have addressed other issues we encountered (e.g., small numbers of studies for osteoporosis models).

These analyses are further constrained by a number of interrelated issues. One factor involves the requirement of constructing a data set of harmonized measures for meta-analyses. While there was conceptual homogeneity in the models and measures, there were some differences in the way in which sexuality and the outcomes were measured. A focus on estimating health inequalities as opposed to prevalence mitigates this concern in part.

A meta-analytic approach can also mean that a more nuanced treatment of the data is lost. Returning to the example of the provision of care, differences in care patterns were expected based on previous findings that LGB people start caring for others *earlier* than heterosexual people (Kneale & French, 2018). In contrast, the meta-analytic models focused on more crude differences in levels of *current* provision of informal care. Overall, the evidence in this paper illuminates that inequalities exist, although in line with critiques around meta-analysis being well equipped to provide a “big fact” but struggling to provide a more “sophisticated answer” (Glass, 2015), the analyses do not indicate when and why inequalities emerge. Other limitations include the small sample sizes within studies, and different study designs and measures preventing raw survey data being combined directly in a one-stage IPD model. Statistical power continued to hinder further exploration of differences in health and care status by age and by sexual orientation. Similarly, while much of this paper is grounded in exploring differences among older LGB people, small sample sizes meant that it reflects the experience of those aged 50+, as opposed to an older threshold. Finally, the absence of transgender people from the analyses was unavoidable due to limitations in the available data.

## Conclusions

While these analyses are not the first to attempt to understand the extent of health inequalities among sexual minorities using individual-level data (Semlyen, King, Varney, & Hagger-Johnson, 2016), these analyses are the first incorporate data from such a wide breadth of data sources, to examine a breadth of indicators, and to focus on people aged 50 and older. Our largest model incorporated data from 25 different data sources and provided an unparalleled sample size (over 2,500 LGB men and women) to enable understanding the extent of sexuality-based health inequalities in later life. The approach of systematically exploring the UKDS uncovered almost 30 different data sources available. Individually, these studies were generally underpowered and no sexuality-based inequality would be detected, although when synthesized the data allowed for the detection of inequalities in self-rated health, long-term illness, smoking, suicide attempts, and life satisfaction, demonstrating the benefits of the IPD meta-analysis approach. A similar framework could be applied to examine inequalities in later life among other minority groups, such as Black and Minority Ethnic groups.

The findings here corroborate the minority stress theory (Frost et al., 2015; Meyer, 1995), but also generate new questions for researchers around when and why these inequalities emerge, and the findings here provide a starting point for these investigations. Drawing on models such as the Health Equity Promotion Model (Fredriksen-Goldsen et al., 2014), that explicitly incorporate notions of the life course, could help in structuring future analyses. These should focus on examining when the inequalities in health uncovered here begin to emerge, and how social position and context shape pathways to health equity and health adversity. Such investigations are, however, dependent on the collection or existence of longitudinal data sources, although only two of the studies included in the present study were of a longitudinal design. Such questions should instead be investigated through ambitious multimethod programs. However, unlike the United States, where substantial investments have been made to understand sexual minority health through the collection of new data across generations (Meyer et al., 2020), the United Kingdom is lacking such investment in LGB health data. The absence of theory-driven data collection in the United Kingdom means that, while we now know where sexuality-based inequalities are broadly located, there remains considerable work to unpack diverse “lumped” categories such as “LGB,” and to understand how decision-makers and practitioners can best support well-being somewhere over the rainbow.

## Supplementary Material

gbaa071_suppl_Supplementary_Material

## References

[CIT0001] Addis S., Davies M., Greene G., MacBride-Stewart S., & Shepherd M (2009). The health, social care and housing needs of lesbian, gay, bisexual and transgender older people: A review of the literature. Health & Social Care in the Community, 17(6), 647–658. doi:10.1111/j.1365-2524.2009.00866.x 19519872

[CIT0002] Almack K., & King A (2019). Lesbian, gay, bisexual, and trans aging in a UK context: Critical observations of recent research literature. The International Journal of Aging and Human Development, 89(1), 93–107. doi:10.1177/0091415019836921 30897923

[CIT0003] Bartley M., & Plewis I (2002). Accumulated labour market disadvantage and limiting long-term illness: Data from the 1971–1991 Office for National Statistics’ Longitudinal Study. International Journal of Epidemiology, 31(2), 336–341. doi:10.1093/ije/31.2.336 11980794

[CIT0004] Bentham G., Eimermann J., Haynes R., Lovett A., & Brainard J (1995). Limiting long-term illness and its associations with mortality and indicators of social deprivation. Journal of Epidemiology & Community Health, 49(Suppl. 2), S57–S64. doi:10.1136/jech.49.suppl_2.s57 8594136 PMC1060878

[CIT0005] Bernstein M (2011). United States: Multi-institutional politics, social movements and the state. In Tremblay M., Patternotte D., & Johnson C. (Eds.), The lesbian and gay movement and the state: Comparative insights into a transformed relationship (pp. 197–212). Farnham, UK: Ashgate.

[CIT0006] Bouman W. P., Claes L., Marshall E., Pinner G. T., Longworth J., Maddox V., . . . Arcelus J (2016). Sociodemographic variables, clinical features, and the role of preassessment cross-sex hormones in older trans people. The Journal of Sexual Medicine, 13(4), 711–719. doi:10.1016/j.jsxm.2016.01.009 26897462

[CIT0007] Brennan D. J., Bauer G. R., Bradley K., & Tran O. V (2017). Methods used and topics addressed in quantitative health research on gay, bisexual and other men who have sex with men: A systematic review of the literature. Journal of Homosexuality, 64(11), 1519–1538. doi:10.1080/00918369.2016.1247537 27754799

[CIT0008] Cohen J (1992). A power primer. Psychological Bulletin, 112(1), 155. doi:10.1037//0033-2909.112.1.155 19565683

[CIT0009] Deeks J., Higgins J., & Altman D (2011). Chapter 9—Analysing data and undertaking meta-analyses. In Higgins J. P. T. & Green S. (Eds.), Cochrane handbook for systematic reviews of interventions version 5.1. 0 [updated March 2011] (Vol. 5, pp. 243–296). Retrieved from The Cochrane Collaboration website: www.handbook.cochrane.org

[CIT0010] Detwiler B. P (2015). Minority stress in the sexual minority older adult population: Exploring the relationships among discrimination, mental health, and quality of life. Bethlehem, PA: Lehigh University.

[CIT0011] Discacciati A., Orsini N., & Greenland S (2015). Approximate Bayesian logistic regression via penalized likelihood by data augmentation. The Stata Journal, 15(3), 712–736. doi:10.1177/1536867X1501500306

[CIT0012] Downes M (2019). Pride, protest and litigation—American gifts to LGBT Britain Retrieved from https://ukhumanrightsblog.com/2019/07/09/pride-protest-and-litigation-american-gifts-to-lgbt-britain/

[CIT0013] Fabbre V., Jen S., & Fredriksen-Goldsen K (2018). The state of theory in LGBTQ aging. Innovation in Aging, 2(Suppl. 1), 65. doi:10.1093/geroni/igy023.244 PMC6760910 30626272

[CIT0014] Firth J., Siddiqi N., Koyanagi A., Siskind D., Rosenbaum S., Galletly C., . . . Carvalho A. F (2019). The Lancet Psychiatry Commission: A blueprint for protecting physical health in people with mental illness. The Lancet Psychiatry, 6(8), 675–712. doi:10.1016/S2215-0366(19)30132-4 31324560

[CIT0015] Fredriksen-Goldsen K. I., Emlet C. A., Kim H.-J., Muraco A., Erosheva E. A., Goldsen J., & Hoy-Ellis C. P (2013). The physical and mental health of lesbian, gay male, and bisexual (LGB) older adults: The role of key health indicators and risk and protective factors. The Gerontologist, 53(4), 664–675. doi:10.1093/geront/gns123 23034470 PMC3709843

[CIT0016] Fredriksen-Goldsen K. I., Simoni J. M., Kim H.-J., Lehavot K., Walters K. L., Yang J., . . . Muraco A (2014). The health equity promotion model: Reconceptualization of lesbian, gay, bisexual, and transgender (LGBT) health disparities. American Journal of Orthopsychiatry, 84(6), 653. doi:10.1037/ort0000030 25545433 PMC4350932

[CIT0017] Frost D. M., Lehavot K., & Meyer I. H (2015). Minority stress and physical health among sexual minority individuals. Journal of Behavioral Medicine, 38(1), 1–8. doi:10.1007/s10865-013-9523-8 23864353 PMC3895416

[CIT0018] Glass G. V (2015). Meta‐analysis at middle age: A personal history. Research Synthesis Methods, 6(3), 221–231. doi:10.1002/jrsm.1133 26355796

[CIT0019] Greenland S., Mansournia M. A., & Altman D. G (2016). Sparse data bias: A problem hiding in plain sight. BMJ (Clinical Research Ed.), 352, i1981. doi:10.1136/bmj.i1981 27121591

[CIT0020] Guasp A (2011). Lesbian, gay and bisexual people later in life. London: Stonewall.

[CIT0021] Harris R. J., Deeks J. J., Altman D. G., Bradburn M. J., Harbord R. M., & Sterne J. A (2008). Metan: Fixed-and random-effects meta-analysis. The Stata Journal, 8(1), 3–28. doi:10.1177/1536867X0800800102

[CIT0022] Higgins J. P. T., & Green S (2011). Cochrane handbook for systematic reviews of interventions (Vol. 5.1.0). Chichester: Wiley-Blackwell.

[CIT0023] Hoy‐Ellis C. P., & Fredriksen‐Goldsen K. I (2017). Depression among transgender older adults: General and minority stress. American Journal of Community Psychology, 59(3–4), 295–305. doi:10.1002/ajcp.12138 28369987 PMC5474152

[CIT0024] Idler E. L., & Benyamini Y (1997). Self-rated health and mortality: A review of twenty-seven community studies. Journal of Health and Social Behavior, 38(1), 21–37. Retrieved from https://www.ncbi.nlm.nih.gov/pubmed/9097506 9097506

[CIT0025] Jagger C., Matthews F. E., Wohland P., Fouweather T., Stephan B. C., Robinson L., . . Medical Research Council Cognitive Function and Ageing Collaboration. (2016). A comparison of health expectancies over two decades in England: Results of the Cognitive Function and Ageing Study I and II. The Lancet, 387(10020), 779–786. doi:10.1016/S0140-6736(15)00947-2 PMC4761658 26680218

[CIT0026] Joloza T., Evans J., O’Brien R., & Potter-Collins A (2010). Measuring sexual identity: an evaluation report. Newport: Office for National Statistics.

[CIT0027] Keleher A., & Smith E. R (2012). Growing support for gay and lesbian equality since 1990. Journal of Homosexuality, 59(9), 1307–1326. doi:10.1080/00918369.2012.720540 23101499

[CIT0028] Kneale D., & French R (2018). Examining life course trajectories of lesbian, gay and bisexual people in England—Exploring convergence and divergence among a heterogeneous population of older people. Longitudinal and Life Course Studies, 9(2), 226–244. doi:10.14301/llcs.v9i2.425 30930964 PMC6436696

[CIT0029] Kneale D., French R., & Thomas J (2018). Inequalities in older LGBT people’s health and care needs in the UK: Protocol for an Individual Participant Data meta-analysis. Retrieved from https://eppi.ioe.ac.uk/cms/LinkClick.aspx?fileticket=rP5hcAl03PQ%3D&tabid=3691&portalid=0&mid=7369

[CIT0030] Kneale D., Henley J., Thomas J., & French R (2019). Inequalities in older LGBT people’s health and care needs in the United Kingdom: A systematic scoping review. Ageing & Society, 1–23. doi:10.1017/S0144686X19001326 34531622 PMC8423450

[CIT0031] Kneale D., Sholl P., Sherwood C., & Faulkner J (2014). Ageing and lesbian, gay and bisexual relationships. Working With Older People, 18(3), 142–151. doi:10.1108/WWOP-06-2014-0015

[CIT0032] Kollman K., & Waites M (2011). United Kingdom: Changing political opportunity structures, policy successes and continuing challenges for lesbian, gay and bisexual movements. In Tremblay M., Patternotte D., & Johnson C. (Eds.), The lesbian and gay movement and the state: Comparative insights into a transformed relationship (pp. 181–196). Farnham, UK: Ashgate.

[CIT0033] Leibel K., Lee J. G., Goldstein A. O., & Ranney L. M (2011). Barring intervention? Lesbian and gay bars as an underutilized venue for tobacco interventions. Nicotine & Tobacco Research, 13(7), 507–511. doi:10.1093/ntr/ntr065 21498874

[CIT0034] McParland J., & Camic P. M (2016). Psychosocial factors and ageing in older lesbian, gay and bisexual people: A systematic review of the literature. Journal of Clinical Nursing, 25(23–24), 3415–3437. doi:10.1111/jocn.13251 27167408

[CIT0035] Meyer I. H (1995). Minority stress and mental health in gay men. Journal of Health and Social Behavior, 36(1), 38–56. Retrieved from https://www.ncbi.nlm.nih.gov/pubmed/7738327 7738327

[CIT0036] Meyer I. H (2003). Prejudice, social stress, and mental health in lesbian, gay, and bisexual populations: Conceptual issues and research evidence. Psychological Bulletin, 129(5), 674–697. doi:10.1037/0033-2909.129.5.674 12956539 PMC2072932

[CIT0037] Meyer, I. H., Marken, S., Russell, S. T., Frost, D. M., Wilson, B. D. (2020). An innovative approach to the design of a national probability sample of sexual minority adults. LGBT health, 7(2), 101–108. 10.1089/lgbt.2019.0145 PMC7138603 32130087

[CIT0038] Mossey J. M., & Shapiro E (1982). Self-rated health: A predictor of mortality among the elderly. American Journal of Public Health, 72(8), 800–808. doi:10.2105/ajph.72.8.800 7091475 PMC1650365

[CIT0039] Office for National Statistics. (2016). Social Surveys Division, Integrated household survey 2009–2014: Secure access [computer file]. Colchester, Essex: UK Data Archive. SN 8075. doi:10.5255/UKDA-SN-8075-1

[CIT0040] Owen G., & Catalan J (2012). “We never expected this to happen”: Narratives of ageing with HIV among gay men living in London, UK. Culture, Health and Sexuality, 14(1), 59–72. doi:10.1080/13691058.2011.621449 22077645

[CIT0041] Park A., & Rhead R (2013). Personal relationships: Homosexuality. In Park A., Bryson C., Clery E., Curtice J., & Phillips M. (Eds.), British social attitudes: The 30th report (pp. 1–32). London: NatCen Social Research.

[CIT0042] Potter C., Bamford S., & Kneale D (2011). Bridging the gap: Exploring the potential for bringing older and younger LGBT people together. Retrieved from https://ilcuk.org.uk/bridging-the-gap-exploring-the-potential-for-bringing-older-and-younger-lgbt-people-together/.

[CIT0043] Public Health England. (2017). Chapter 1: Life expectancy and healthy life expectancy. In PHE (Ed.), Health profile for England: 2017. London: Public Health England.

[CIT0044] Riley R. D., Lambert P. C., & Abo-Zaid G (2010). Meta-analysis of individual participant data: Rationale, conduct, and reporting. BMJ (Clinical Research Ed.), 340, c221. doi:10.1136/bmj.c221 20139215

[CIT0045] Rizer A. M., Mauery D. R., Haynes S. G., Couser B., & Gruman C (2015). Challenges in intervention research for lesbian and bisexual women. LGBT Health, 2(2), 105–112. doi:10.1089/lgbt.2014.0122 26790115

[CIT0046] Rodger A. J., Cambiano V., Bruun T., Vernazza P., Collins S., Degen O., . . . Beloukas A (2019). Risk of HIV transmission through condomless sex in serodifferent gay couples with the HIV-positive partner taking suppressive antiretroviral therapy (PARTNER): Final results of a multicentre, prospective, observational study. The Lancet. Advance online publication. doi:10.1016/s0140-6736(19)30418-0 PMC6584382 31056293

[CIT0047] Sedlak C. A., Roller C. G., van Dulmen M., Alharbi H. A., Sanata J. D., Leifson M. A., . . . Doheny M. O (2017). Transgender individuals and osteoporosis prevention. Orthopedic Nursing, 36(4), 259–268. doi:10.1097/NOR.0000000000000364 28737632

[CIT0048] Semlyen J., King M., Varney J., & Hagger-Johnson G (2016). Sexual orientation and symptoms of common mental disorder or low wellbeing: Combined meta-analysis of 12 UK population health surveys. BMC Psychiatry, 16, 67. doi:10.1186/s12888-016-0767-z 27009565 PMC4806482

[CIT0049] Stewart L. A., Clarke M., Rovers M., Riley R. D., Simmonds M., Stewart G., . . . Tierney J. F.; PRISMA-IPD Development Group (2015). Preferred reporting items for systematic review and meta-analyses of individual participant data: The PRISMA-IPD statement. JAMA, 313(16), 1657–1665. doi:10.1001/jama.2015.3656 25919529

[CIT0050] Vittinghoff E., & McCulloch C. E (2007). Relaxing the rule of ten events per variable in logistic and Cox regression. American Journal of Epidemiology, 165(6), 710–718. doi:10.1093/aje/kwk052 17182981

[CIT0051] Wallace M. G (2018). Black history month: A closer look at the minority stress model and older adult sexual minorities. Retrieved from https://www.diverseelders.org/2018/02/12/black-history-month- a-closer-look-at-the-minority-stress-model-and-older-adult-sexual-minorities/.

[CIT0052] Westwood S., Willis P., Fish J., Hafford-Letchfield T., Semlyen J., King A., . . . Becares L (2020). Older LGBT+ health inequalities in the UK: Setting a research agenda. Journal of Epidemiology and Community Health, 74(5), 408–411. doi:10.1136/jech-2019-213068 32086374

[CIT0053] Wierckx K., Mueller S., Weyers S., Van Caenegem E., Roef G., Heylens G., & T’Sjoen G (2012). Long-term evaluation of cross-sex hormone treatment in transsexual persons. The Journal of Sexual Medicine, 9(10), 2641–2651. doi:10.1111/j.1743-6109.2012.02876.x 22906135

