# Supplementary material for: Inequalities in Health and Care Among Lesbian, Gay, and Bisexual People Aged 50 and Older in the United Kingdom: A Systematic Review and Meta-analysis of Sources of Individual Participant Data
Source: J Gerontol B Psychol Sci Soc Sci. 2020 May 31;75(8):1758–71. doi: 10.1093/geronb/gbaa071 (PMC7489108; doi:10.1093/geronb/gbaa071)
Supplement: gbaa071_suppl_Supplementary_Material [file gbaa071_suppl_supplementary_material.docx]

Inequalities in health and care among Lesbian, Gay and Bisexual people aged 50 and over in the United Kingdom: A meta-analysis of individual participant data

Contents

[Search details 3](#_Toc34212926)

[Appendix 1 - Prisma Flow of Records through the review 4](#_Toc34212927)

[Appendix 2 - Prisma Checklist for IPD analyses 5](#_Toc34212928)

[Appendix 3 – Forest Plot and further details for Self-rated health 9](#_Toc34212929)

[Overall – unadjusted estimates with totals in brackets 9](#_Toc34212930)

[Overall –adjusted estimates with totals in brackets 10](#_Toc34212931)

[Males –unadjusted estimates with totals in brackets 11](#_Toc34212932)

[Males –adjusted estimates with totals in brackets 12](#_Toc34212933)

[Females –unadjusted estimates with totals in brackets 13](#_Toc34212934)

[Females –adjusted estimates with totals in brackets 14](#_Toc34212935)

[Further data notes – Self-Rated Health 14](#_Toc34212936)

[Appendix 4 – Forest Plots and further details for limitations due to health or illness 16](#_Toc34212937)

[Overall – unadjusted estimates with totals in brackets 16](#_Toc34212938)

[Overall –adjusted estimates with totals in brackets 16](#_Toc34212939)

[Males –unadjusted estimates with totals in brackets 18](#_Toc34212940)

[Males –adjusted estimates with totals in brackets 19](#_Toc34212941)

[Females –unadjusted estimates with totals in brackets 20](#_Toc34212942)

[Females –adjusted estimates with totals in brackets 21](#_Toc34212943)

[Further data notes 21](#_Toc34212944)

[Appendix 5 – Forest Plots and further details for Long-term Illness or Health Conditions 23](#_Toc34212945)

[Overall – unadjusted estimates with totals in brackets 23](#_Toc34212946)

[Overall –adjusted estimates with totals in brackets 24](#_Toc34212947)

[Males –unadjusted estimates with totals in brackets 25](#_Toc34212948)

[Males –adjusted estimates with totals in brackets 26](#_Toc34212949)

[Females –unadjusted estimates with totals in brackets 27](#_Toc34212950)

[Females –adjusted estimates with totals in brackets 28](#_Toc34212951)

[Further data notes 28](#_Toc34212952)

[Appendix 6 – Forest Plots and further details for Low Life Satisfaction 30](#_Toc34212953)

[Overall – unadjusted estimates with totals in brackets 30](#_Toc34212954)

[Overall –adjusted estimates with totals in brackets 31](#_Toc34212955)

[Males –unadjusted estimates with totals in brackets 32](#_Toc34212956)

[Males –adjusted estimates with totals in brackets 33](#_Toc34212957)

[Females –unadjusted estimates with totals in brackets 34](#_Toc34212958)

[Females –adjusted estimates with totals in brackets 35](#_Toc34212959)

[Further data notes 35](#_Toc34212960)

[Appendix 7 – Forest Plots and further details for Lifetime Suicide Attempts 37](#_Toc34212961)

[Overall – unadjusted estimates with totals in brackets 37](#_Toc34212962)

[Overall –adjusted estimates with totals in brackets 37](#_Toc34212963)

[Males –unadjusted estimates with totals in brackets 39](#_Toc34212964)

[Males –adjusted estimates with totals in brackets 40](#_Toc34212965)

[Females –unadjusted estimates with totals in brackets 41](#_Toc34212966)

[Females –adjusted estimates with totals in brackets 42](#_Toc34212967)

[Further data notes 42](#_Toc34212968)

[Appendix 8 – Forest Plots and further details for Provision of Care 43](#_Toc34212969)

[Overall – unadjusted estimates with totals in brackets 43](#_Toc34212970)

[Overall –adjusted estimates with totals in brackets 43](#_Toc34212971)

[Males –unadjusted estimates with totals in brackets 45](#_Toc34212972)

[Males –adjusted estimates with totals in brackets 46](#_Toc34212973)

[Females –unadjusted estimates with totals in brackets 47](#_Toc34212974)

[Females –adjusted estimates with totals in brackets 48](#_Toc34212975)

[Further data notes 48](#_Toc34212976)

[Appendix 9 – Forest Plots and further details for Heavy Drinking 50](#_Toc34212977)

[Overall – unadjusted estimates with totals in brackets 50](#_Toc34212978)

[Overall –adjusted estimates with totals in brackets 51](#_Toc34212979)

[Males –unadjusted estimates with totals in brackets 52](#_Toc34212980)

[Males –adjusted estimates with totals in brackets 53](#_Toc34212981)

[Females –unadjusted estimates with totals in brackets 54](#_Toc34212982)

[Females –adjusted estimates with totals in brackets 55](#_Toc34212983)

[Further data notes 55](#_Toc34212984)

[Appendix 10 – Forest Plots and further details for Current Smoking 56](#_Toc34212985)

[Overall – unadjusted estimates with totals in brackets 57](#_Toc34212986)

[Overall –adjusted estimates with totals in brackets 58](#_Toc34212987)

[Males –unadjusted estimates with totals in brackets 59](#_Toc34212988)

[Males –adjusted estimates with totals in brackets 60](#_Toc34212989)

[Females –unadjusted estimates with totals in brackets 61](#_Toc34212990)

[Females –adjusted estimates with totals in brackets 62](#_Toc34212991)

[Further data notes 62](#_Toc34212992)

# Search details

The following terms were used to search within the repository: “sexual identity”, “sexual orientation”, “same sex”, “gender identity”, “lesbian”, “gay”, “bisexual”, “transgender”, “transsexual”, “transvestite”, “queer”, “LGBT”, “LGB”, “homosexuality”, “sexual attraction”, “sexual experience”, “sexual desire”. Due to the repository not having been designed for the purposes of conducting a systematic review, simple search terms reflecting sexuality alone were used, and all records screened initially based on information contained within the abstract (equivalent to title and abstract screening), and where appropriate the study documentation including the original questionnaire to establish relevance (equivalent to full-text screening). Because the repository does not allow for dataset records to be exported out of the system, the search instead was conducted following the principles laid out by Stansfield, Dickson, and Bangpan (2016) on conducting website searching including (i) detailing the rationale for the search; (ii) utilising the resources in a consistent way; and (iii) recordkeeping of the searches executed (Stansfield et al., 2016). One of our limitations of this approach is that, unlike when screening for published studies, fewer details outlining the flow of studies through a review can be understood and recorded.

# Appendix 1 - Prisma Flow of Records through the review

Figure 1: Flow of data sources through the review

# Appendix 2 - Prisma Checklist for IPD analyses

**PRISMA-IPD Checklist of items to include when reporting a systematic review and meta-analysis of individual participant data (IPD)**

| **PRISMA-IPD**  **Section/topic** | **Item No** | **Checklist item** | **Reported on page** |
| --- | --- | --- | --- |
| **Title** | | | |
| Title | 1 | Identify the report as a systematic review and meta-analysis of individual participant data. | p1 |
| Abstract | | | |
| Structured summary | 2 | Provide a structured summary including as applicable: | p1 |
|  |  | **Background**: state research question and main objectives, with information on participants, interventions, comparators and outcomes. |  |
|  |  | **Methods**: report eligibility criteria; data sources including dates of last bibliographic search or elicitation, noting that IPD were sought; methods of assessing risk of bias. |  |
|  |  | **Results**: provide number and type of studies and participants identified and number (%) obtained; summary effect estimates for main outcomes (benefits and harms) with confidence intervals and measures of statistical heterogeneity. Describe the direction and size of summary effects in terms meaningful to those who would put findings into practice. |  |
|  |  | **Discussion:** state main strengths and limitations of the evidence, general interpretation of the results and any important implications. |  |
|  |  | **Other:** report primary funding source, registration number and registry name for the systematic review and IPD meta-analysis. |  |
| Introduction | | | |
| Rationale | 3 | Describe the rationale for the review in the context of what is already known. | p4-7 |
| Objectives | 4 | Provide an explicit statement of the questions being addressed with reference, as applicable, to participants, interventions, comparisons, outcomes and study design (PICOS). Include any hypotheses that relate to particular types of participant-level subgroups. | p8 |
| Methods | | | |
| Protocol and registration | 5 | Indicate if a protocol exists and where it can be accessed. If available, provide registration information including registration number and registry name. Provide publication details, if applicable. | p7 |
| Eligibility criteria | 6 | Specify inclusion and exclusion criteria including those relating to participants, interventions, comparisons, outcomes, study design and characteristics (e.g. years when conducted, required minimum follow-up). Note whether these were applied at the study or individual level i.e. whether eligible participants were included (and ineligible participants excluded) from a study that included a wider population than specified by the review inclusion criteria. The rationale for criteria should be stated. | p8 |
| Identifying studies - information sources | 7 | Describe all methods of identifying published and unpublished studies including, as applicable: which bibliographic databases were searched with dates of coverage; details of any hand searching including of conference proceedings; use of study registers and agency or company databases; contact with the original research team and experts in the field; open adverts and surveys. Give the date of last search or elicitation. | p3 Supplementary materials |
| Identifying studies - search | 8 | Present the full electronic search strategy for at least one database, including any limits used, such that it could be repeated. | p3 |
| Study selection processes | 9 | State the process for determining which studies were eligible for inclusion. | p8 |
| Data collection processes | 10 | Describe how IPD were requested, collected and managed, including any processes for querying and confirming data with investigators. If IPD were not sought from any eligible study, the reason for this should be stated (for each such study). | p3-p4 Supplementary materials; p8; p12 |
|  |  | If applicable, describe how any studies for which IPD were not available were dealt with. This should include whether, how and what aggregate data were sought or extracted from study reports and publications (such as extracting data independently in duplicate) and any processes for obtaining and confirming these data with investigators. |  |
| Data items | 11 | Describe how the information and variables to be collected were chosen. List and define all study level and participant level data that were sought, including baseline and follow-up information. If applicable, describe methods of standardising or translating variables within the IPD datasets to ensure common scales or measurements across studies. | p8 |
| IPD integrity | A1 | Describe what aspects of IPD were subject to data checking (such as sequence generation, data consistency and completeness, baseline imbalance) and how this was done. | Observational data: p9 |
| Risk of bias assessment in individual studies. | 12 | Describe methods used to assess risk of bias in the individual studies and whether this was applied separately for each outcome. If applicable, describe how findings of IPD checking were used to inform the assessment. Report if and how risk of bias assessment was used in any data synthesis. | N/A |
| Specification of outcomes and effect measures | 13 | State all treatment comparisons of interests. State all outcomes addressed and define them in detail. State whether they were pre-specified for the review and, if applicable, whether they were primary/main or secondary/additional outcomes. Give the principal measures of effect (such as risk ratio, hazard ratio, difference in means) used for each outcome. | p8; p12 |
| Synthesis methods | 14 | Describe the meta-analysis methods used to synthesise IPD. Specify any statistical methods and models used. Issues should include (but are not restricted to):   - Use of a one-stage or two-stage approach. - How effect estimates were generated separately within each study and combined across studies (where applicable). - Specification of one-stage models (where applicable) including how clustering of patients within studies was accounted for. - Use of fixed or random effects models and any other model assumptions, such as proportional hazards. - How (summary) survival curves were generated (where applicable). - Methods for quantifying statistical heterogeneity (such as I^2^ and τ^2^). - How studies providing IPD and not providing IPD were analysed together (where applicable). - How missing data within the IPD were dealt with (where applicable). | p9-p10 |
| Exploration of variation in effects | A2 | If applicable, describe any methods used to explore variation in effects by study or participant level characteristics (such as estimation of interactions between effect and covariates). State all participant-level characteristics that were analysed as potential effect modifiers, and whether these were pre-specified. | p10-p11 |
| Risk of bias across studies | 15 | Specify any assessment of risk of bias relating to the accumulated body of evidence, including any pertaining to not obtaining IPD for particular studies, outcomes or other variables. | N/A |
| Additional analyses | 16 | Describe methods of any additional analyses, including sensitivity analyses. State which of these were pre-specified. | p10-p11 |
| Results | | | |
| Study selection and IPD obtained | 17 | Give numbers of studies screened, assessed for eligibility, and included in the systematic review with reasons for exclusions at each stage. Indicate the number of studies and participants for which IPD were sought and for which IPD were obtained. For those studies where IPD were not available, give the numbers of studies and participants for which aggregate data were available. Report reasons for non-availability of IPD. Include a flow diagram. | P4 supplementary materials |
| Study characteristics | 18 | For each study, present information on key study and participant characteristics (such as description of interventions, numbers of participants, demographic data, unavailability of outcomes, funding source, and if applicable duration of follow-up). Provide (main) citations for each study. Where applicable, also report similar study characteristics for any studies not providing IPD. | p10; p14-15 |
| IPD integrity | A3 | Report any important issues identified in checking IPD or state that there were none. | None |
| Risk of bias within studies | 19 | Present data on risk of bias assessments. If applicable, describe whether data checking led to the up-weighting or down-weighting of these assessments. Consider how any potential bias impacts on the robustness of meta-analysis conclusions. | N/A |
| Results of individual studies | 20 | For each comparison and for each main outcome (benefit or harm), for each individual study report the number of eligible participants for which data were obtained and show simple summary data for each intervention group (including, where applicable, the number of events), effect estimates and confidence intervals. These may be tabulated or included on a forest plot. | p14-15 |
| Results of syntheses | 21 | Present summary effects for each meta-analysis undertaken, including confidence intervals and measures of statistical heterogeneity. State whether the analysis was pre-specified, and report the numbers of studies and participants and, where applicable, the number of events on which it is based. | p16-p21 |
|  |  | When exploring variation in effects due to patient or study characteristics, present summary interaction estimates for each characteristic examined, including confidence intervals and measures of statistical heterogeneity. State whether the analysis was pre-specified. State whether any interaction is consistent across trials. |  |
|  |  | Provide a description of the direction and size of effect in terms meaningful to those who would put findings into practice. |  |
| Risk of bias across studies | 22 | Present results of any assessment of risk of bias relating to the accumulated body of evidence, including any pertaining to the availability and representativeness of available studies, outcomes or other variables. | N/A |
| Additional analyses | 23 | Give results of any additional analyses (e.g. sensitivity analyses). If applicable, this should also include any analyses that incorporate aggregate data for studies that do not have IPD. If applicable, summarise the main meta-analysis results following the inclusion or exclusion of studies for which IPD were not available. | p16-p21 |
| Discussion | | | |
| Summary of evidence | 24 | Summarise the main findings, including the strength of evidence for each main outcome. | p16-p21 |
| Strengths and limitations | 25 | Discuss any important strengths and limitations of the evidence including the benefits of access to IPD and any limitations arising from IPD that were not available. | p23-p24 |
| Conclusions | 26 | Provide a general interpretation of the findings in the context of other evidence. | p21-p23 |
| Implications | A4 | Consider relevance to key groups (such as policy makers, service providers and service users). Consider implications for future research. | p24-p26 |
| **Funding** | | | |
| Funding | 27 | Describe sources of funding and other support (such as supply of IPD), and the role in the systematic review of those providing such support. |  |

**A1 – A3 denote new items that are additional to standard PRISMA items. A4 has been created as a result of re-arranging content of the standard PRISMA statement to suit the way that systematic review IPD meta-analyses are reported.**

© Reproduced with permission of the PRISMA IPD Group, which encourages sharing and reuse for non-commercial purposes

# Appendix 3 – Forest Plot and further details for Self-rated health

Please note that the citation for IHS 2014 data is: Office for National Statistics. Social Surveys Division, *Integrated Household Survey 2009-2014: Secure Access* [computer file]. Colchester, Essex: UK Data Archive [distributor], November 2016. SN 8075, http://dx.doi.org/10.5255/UKDA-SN-8075-1

## Overall – unadjusted estimates with totals in brackets


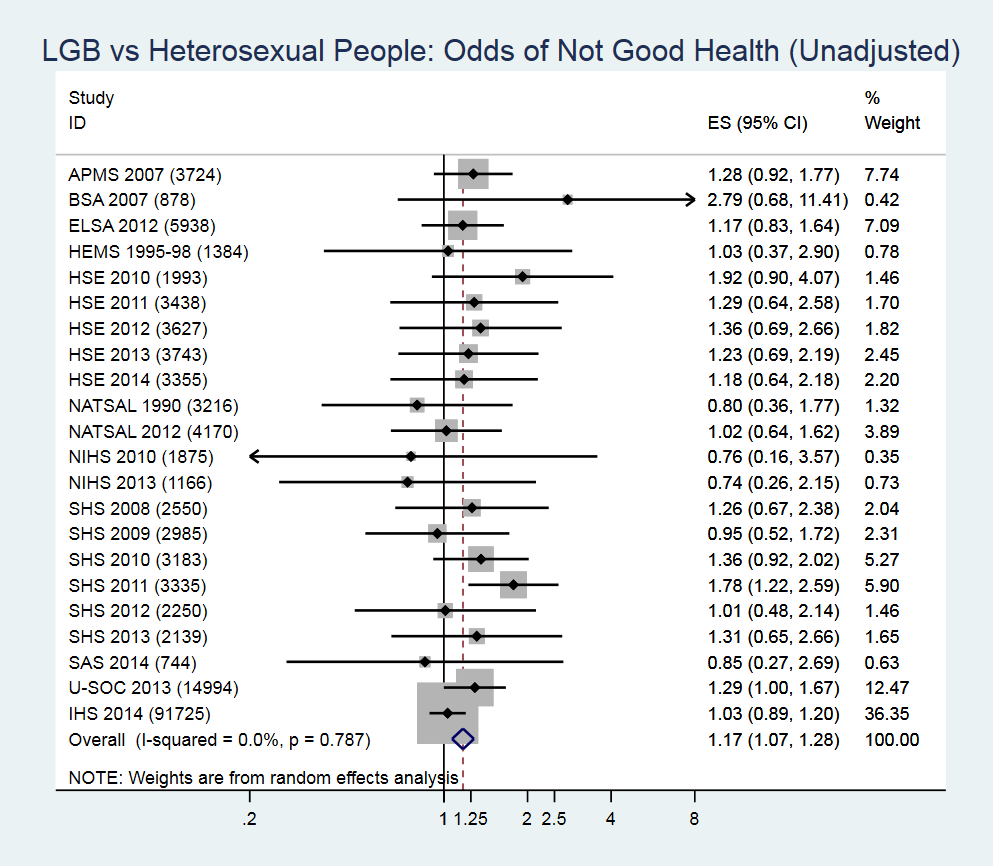


## Overall –adjusted estimates with totals in brackets


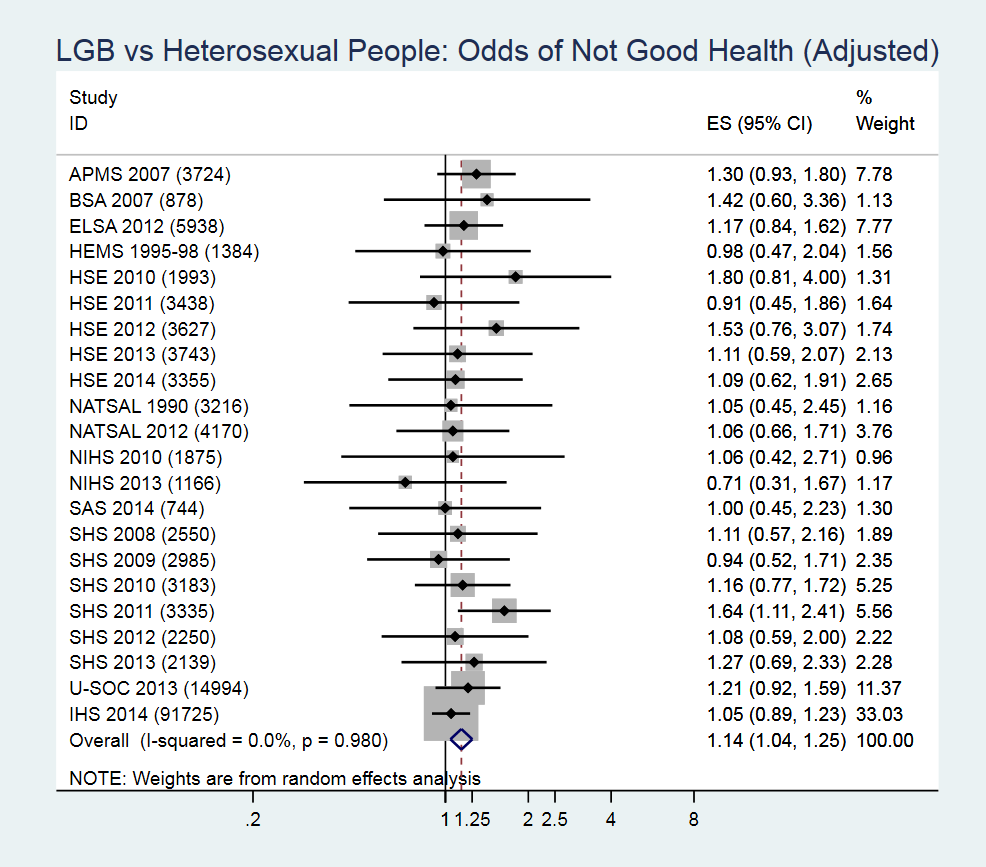


## Males –unadjusted estimates with totals in brackets


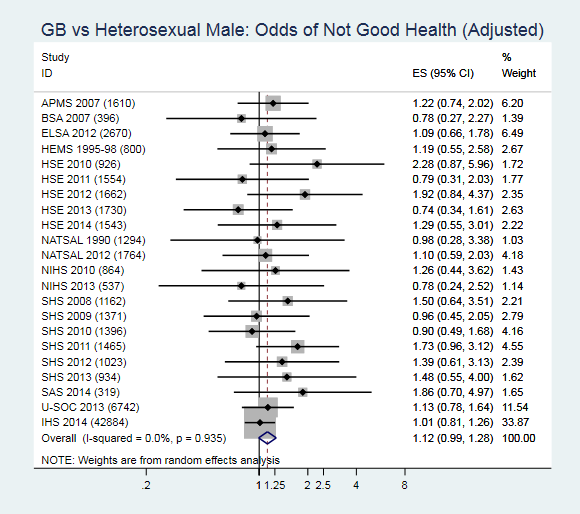


## Males –adjusted estimates with totals in brackets


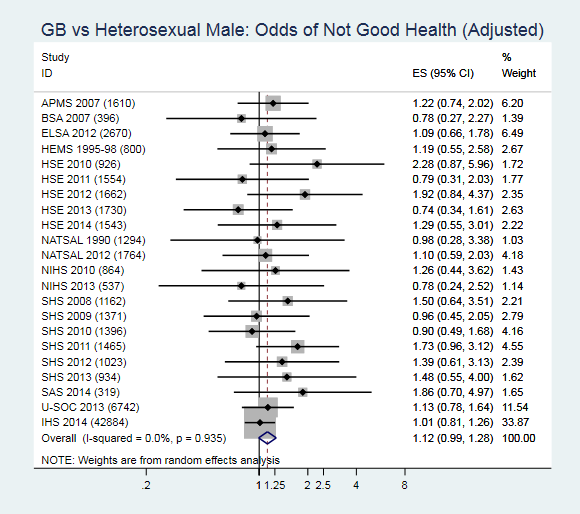


## Females –unadjusted estimates with totals in brackets

## Females –adjusted estimates with totals in brackets

## Further data notes – Self-Rated Health

| Data source abbreviation | Question Details | Model details |
| --- | --- | --- |
| APMS 2007 | Scale: 5 Cat (E,VG,G,F,P) Threshold: Fair/Poor |  |
| BSA 2007 | Scale: 5 Cat (E,VG,G,F,P) Threshold: Fair/Poor | Approximate Bayesian Logistic models used for adjusted estimates for overall and sex-specific model |
| ELSA 2012 | Scale: 5 Cat (E,VG,G,F,P) Threshold: Fair/Poor |  |
| HEMS 1995-98 | Scale: 5 Cat (VG,G,F,B,VB) Threshold: Fair/Bad/Very Bad | Approximate Bayesian Logistic used for unadjusted and adjusted female models |
| HSE 2010 | Scale: 5 Cat (VG,G,F,B,VB) Threshold: Fair/Bad/Very Bad | Approximate Bayesian Logistic models used for adjusted estimates for female model |
| HSE 2011 | Scale: 5 Cat (VG,G,F,B,VB) Threshold: Fair/Bad/Very Bad | Approximate Bayesian Logistic models used for adjusted estimates for female model |
| HSE 2012 | Scale: 5 Cat (VG,G,F,B,VB) Threshold: Fair/Bad/Very Bad | Approximate Bayesian Logistic models used for adjusted estimates for female model |
| HSE 2013 | Scale: 5 Cat (VG,G,F,B,VB) Threshold: Fair/Bad/Very Bad |  |
| HSE 2014 | Scale: 5 Cat (VG,G,F,B,VB) Threshold: Fair/Bad/Very Bad |  |
| NATSAL 1990 | Scale: 5 Cat (VG, Fairly Good, Average, Rather Poor, Very Poor) Threshold: Rather Poor, Very Poor |  |
| NATSAL 2012 | Scale: 5 Cat (VG,G,F,B,VB) Threshold: Bad/Very Bad |  |
| NIHS 2010 | Scale: 5 Cat (VG,G,F,B,VB) Threshold: Bad/Very Bad | Approximate Bayesian Logistic models used for adjusted estimates for overall and sex-specific model |
| NIHS 2013 | Scale: 5 Cat (VG,G,F,B,VB) Threshold: Bad/Very Bad | Approximate Bayesian Logistic used for unadjusted and adjusted male model |
| SAS 2014 | Scale: 5 Cat (VG,G,F,B,VB) Threshold: Fair/Bad/Very Bad | Approximate Bayesian Logistic models used for adjusted estimates for overall and sex-specific model and female unadjusted model |
| SHS 2008 | Scale: 5 Cat (VG,G,F,B,VB) Threshold: Fair/Bad/Very Bad |  |
| SHS 2009 | Scale: 5 Cat (VG,G,F,B,VB) Threshold: Fair/Bad/Very Bad |  |
| SHS 2010 | Scale: 5 Cat (VG,G,F,B,VB) Threshold: Fair/Bad/Very Bad |  |
| SHS 2011 | Scale: 5 Cat (VG,G,F,B,VB) Threshold: Fair/Bad/Very Bad |  |
| SHS 2012 | Scale: 5 Cat (VG,G,F,B,VB) Threshold: Fair/Bad/Very Bad |  |
| SHS 2013 | Scale: 5 Cat (VG,G,F,B,VB) Threshold: Fair/Bad/Very Bad |  |
| U-SOC 2013 | Scale: 5 Cat (E,VG,G,F,P) Threshold: Fair/Poor |  |
| IHS 2014 | Scale: 5 Cat (VG,G,F,B,VB) Threshold: Fair/Bad/Very Bad |  |

# Appendix 4 – Forest Plots and further details for limitations due to health or illness

## Overall – unadjusted estimates with totals in brackets

## Overall –adjusted estimates with totals in brackets

## Males –unadjusted estimates with totals in brackets

## Males –adjusted estimates with totals in brackets

## Females –unadjusted estimates with totals in brackets

## Females –adjusted estimates with totals in brackets

## Further data notes

| Data source abbreviation | Question details | Model details |
| --- | --- | --- |
| ActPS 2014 | Does you illness or disability limit your activities in any way? |  |
| APMS 2007 | Whether health limits moderate activities. |  |
| ELSA 2012 | Whether long-standing Illness is limiting |  |
| HEMS 1995-98 | Whether had limiting long-standing illness | Approximate Bayesian Logistic models used for adjusted estimates for female model |
| HSE 2010 | Whether had limiting long-lasting illness | Approximate Bayesian Logistic models used for adjusted estimates for female model |
| HSE 2011 | Whether had limiting long-lasting illness | Approximate Bayesian Logistic models used for adjusted estimates for female model |
| HSE 2012 | Whether had limiting long-lasting illness |  |
| HSE 2013 | Whether had limiting long-lasting illness |  |
| HSE 2014 | Whether had limiting long-lasting illness |  |
| NATSAL 2012 | Limiting longstanding illness, disability or infirmity |  |
| NIHS 2010 | Illness or disability limits your activities | Approximate Bayesian Logistic models used for adjusted estimates for female, male and combined models |
| NIHS 2013 | Illness or disability limits your activities | Approximate Bayesian Logistic models used for adjusted estimates for male model |
| ONS OMNIB 2004 | Health problems limiting activity or work? | Unadjusted estimates only used |
| SHS 2008 | Whether activities are limited due to illness |  |
| SHS 2009 | Whether activities are limited due to illness |  |
| SHS 2010 | Whether activities are limited due to illness |  |
| SHS 2011 | Whether activities are limited due to illness |  |
| SHS 2012 | Whether activities are limited due to illness |  |
| SHS 2013 | Whether activities are limited due to illness |  |
| U-SOC 2013 | Whether health limits moderate activities. |  |

# Appendix 5 – Forest Plots and further details for Long-term Illness or Health Conditions

## Overall – unadjusted estimates with totals in brackets

## Overall –adjusted estimates with totals in brackets

## Males –unadjusted estimates with totals in brackets

## Males –adjusted estimates with totals in brackets

## Females –unadjusted estimates with totals in brackets

## Females –adjusted estimates with totals in brackets

## Further data notes

| Data source abbreviation | Question Details | Model details |
| --- | --- | --- |
| ActPS 2014 | Do you have a long-standing illness, disability or infirmity? |  |
| ELSA 2012 | Whether has self-reported long-standing illness |  |
| HEMS 1995-98 | Do you have any long-standing illness? | Approximate Bayesian Logistic models used for adjusted estimates for female model |
| HSE 2010 | Whether have any physical or mental health conditions or illnesses | Approximate Bayesian Logistic models used for adjusted estimates for female model |
| HSE 2011 | Whether have any physical or mental health conditions or illnesses | Approximate Bayesian Logistic models used for adjusted estimates for female model |
| HSE 2012 | Whether have any physical or mental health conditions or illnesses |  |
| HSE 2013 | Whether have any physical or mental health conditions or illnesses |  |
| HSE 2014 | Whether have any physical or mental health conditions or illnesses |  |
| NATSAL 1990 | Experienced 3 month illness in last 5 yrs |  |
| NATSAL 2012 | Longstanding illness, disability  or infirmity |  |
| NIHS 2010 | Long-standing illness, disability or infirmity | Approximate Bayesian Logistic models used for adjusted estimates for female, male and combined models |
| NIHS 2013 | Long-standing illness, disability or infirmity | Approximate Bayesian Logistic models used for adjusted estimates for male model |
| SHS 2008 | Whether has longstanding illness |  |
| SHS 2009 | Whether has longstanding illness |  |
| SHS 2010 | Whether has longstanding illness |  |
| SHS 2011 | Whether has longstanding illness |  |
| SHS 2012 | Whether has longstanding illness |  |
| SHS 2013 | Whether has longstanding illness |  |
| SAS 2014 | Do you have any long-term illnesses, health problems or disabilities? | Approximate Bayesian Logistic models used for adjusted estimates for female, male and combined models |
| SAS 2015 | Do you have any long-term illnesses, health problems or disabilities? | Approximate Bayesian Logistic models used for adjusted estimates for female, male and combined models |
| U-SOC 2013 | Long-standing illness or disability |  |

# Appendix 6 – Forest Plots and further details for Low Life Satisfaction

## Overall – unadjusted estimates with totals in brackets

## Overall –adjusted estimates with totals in brackets

## Males –unadjusted estimates with totals in brackets

## Males –adjusted estimates with totals in brackets

## Females –unadjusted estimates with totals in brackets

## Females –adjusted estimates with totals in brackets

## Further data notes

| Data source abbreviation | Question Details | Model details |
| --- | --- | --- |
| ELSA 2012 | Is the respondent satisfied with his/her life  Scale: 7 Cat (SA, A, SA, NAD, SD, D, SD)  Threshold: Slightly disagree-Strongly disagree |  |
| NIHS 2010 | Is the respondent satisfied with his/her life  Scale: 5 Cat (VS, S, NSD, D, SD)  Threshold: Dissatisfied-Strongly dissatisfied |  |
| NIHS 2013 | Is the respondent satisfied with his/her life  Scale: 5 Cat (VS, S, NSD, D, SD)  Threshold: Dissatisfied-Strongly dissatisfied | Firthlogit models used for adjusted and unadjusted models |
| SHS 2008 | (Partially marked) scale 0-10 from extremely dissatisfied to extremely satisfied.  Threshold: 0-4 |  |
| SHS 2009 | (Partially marked) scale 0-10 from extremely dissatisfied to extremely satisfied.  Threshold: 0-4 |  |
| SHS 2010 | (Partially marked) scale 0-10 from extremely dissatisfied to extremely satisfied.  Threshold: 0-4 |  |
| SHS 2011 | (Partially marked) scale 0-10 from extremely dissatisfied to extremely satisfied.  Threshold: 0-4 |  |
| SHS 2012 | (Partially marked) scale 0-10 from extremely dissatisfied to extremely satisfied.  Threshold: 0-4 |  |
| SHS 2013 | (Partially marked) scale 0-10 from extremely dissatisfied to extremely satisfied.  Threshold: 0-4 | Firthlogit model used for adjusted model for females |
| USOC 2013 | Satisfaction with life overall  Scale: 7 Cat (CD, MD, SD, NDS, SS, MS, CS)  Threshold: Completely dissatisfied to somewhat dissatisfied |  |

# Appendix 7 – Forest Plots and further details for Lifetime Suicide Attempts

## Overall – unadjusted estimates with totals in brackets

## Overall –adjusted estimates with totals in brackets

## Males –unadjusted estimates with totals in brackets

## Males –adjusted estimates with totals in brackets

## Females –unadjusted estimates with totals in brackets

## Females –adjusted estimates with totals in brackets

## Further data notes

| Data source abbreviation | Question Details | Model details |
| --- | --- | --- |
| APMS 2007 | Attempts made at suicide during lifetime |  |
| HSE 2014 | Have you ever made an attempt to take your life, by taking an overdose of tablets or in some other way? | Firthlogit models used for adjusted female model |
| SHS 2008 | Ever made an attempt to take own life? | Firthlogit models used for adjusted male model |
| SHS 2011 | Ever made an attempt to take own life? | Approximate Bayesian Logistic Regression used for adjusted female, male and overall models |

Scottish Health Survey 2009 collected data on suicide attempts only from a subset of survey respondents and data are not included in the analysis due to very low number of LGBT respondents included in the subset which could not be disaggregated by gender; Scottish Health Survey 2013 collected data on suicide attempts only from a subset of survey respondents and not included in the analysis due to very low number of LGBT respondents included in the subset; Scottish Health Survey 2010 collected data on suicide attempts only from a subset of survey respondents and not included in the analysis due to very low number of LGBT respondents included in the subset

# Appendix 8 – Forest Plots and further details for Provision of Care

## Overall – unadjusted estimates with totals in brackets

## Overall –adjusted estimates with totals in brackets

## Males –unadjusted estimates with totals in brackets

## Males –adjusted estimates with totals in brackets

## Females –unadjusted estimates with totals in brackets

## Females –adjusted estimates with totals in brackets

## Further data notes

| Data source abbreviation | Question Details | Model details |
| --- | --- | --- |
| APMS 2007 | Look after family members, friends or others due to their health or disability |  |
| ELSA 2012 | Respondent reports that they looked after her/his spouse/partner; parent; parent-in-law; other relative; friend or neighbour; some other person |  |
| HSE 2011 | Whether provided help to anyone with long-term physical/mental illhealth/ disability | Approximate Bayesian Logistic models used for adjusted estimates for female model |
| HSE 2012 | Whether provided help to anyone with long-term physical/mental illhealth/ disability |  |
| HSE 2013 | Whether provided help to anyone with long-term physical/mental illhealth/ disability |  |
| HSE 2014 | Whether provided help to anyone with long-term physical/mental illhealth/ disability |  |
| NIHS 2010 | Respondent reports caring for someone | Approximate Bayesian Logistic models used for adjusted estimates for female, male and combined models |
| NIHS 2013 | Respondent reports caring for someone | Approximate Bayesian Logistic models used for adjusted estimates for male and model |
| SHS 2008 | Do you provide any regular help or care for any sick, disabled or frail person? |  |
| SHS 2009 | Do you provide any regular help or care for any sick, disabled or frail person? |  |
| SHS 2010 | Do you provide any regular help or care for any sick, disabled or frail person? |  |
| SHS 2011 | Do you provide any regular help or care for any sick, disabled or frail person? |  |
| SHS 2012 | Do you provide any regular help or care for any sick, disabled or frail person? |  |
| SHS 2013 | Do you provide any regular help or care for any sick, disabled or frail person? |  |
| U-SOC 2013 | Cares for handicapped/other person needing help in the household or outside the household |  |

# Appendix 9 – Forest Plots and further details for Heavy Drinking

## Overall – unadjusted estimates with totals in brackets


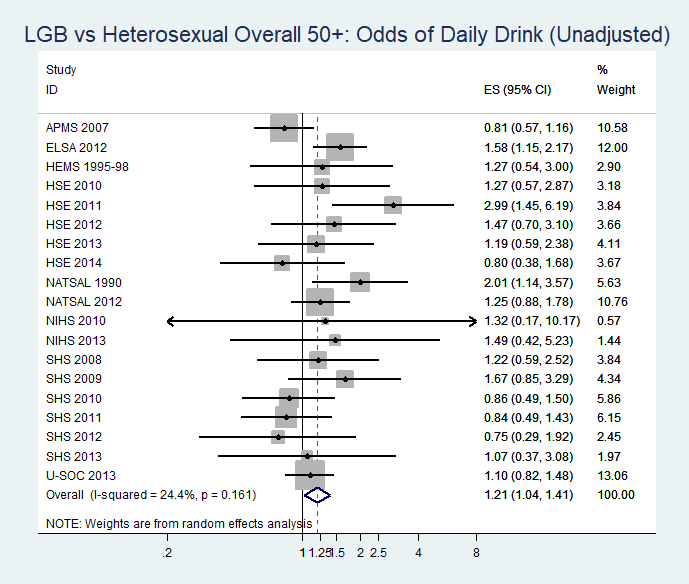


## Overall –adjusted estimates with totals in brackets


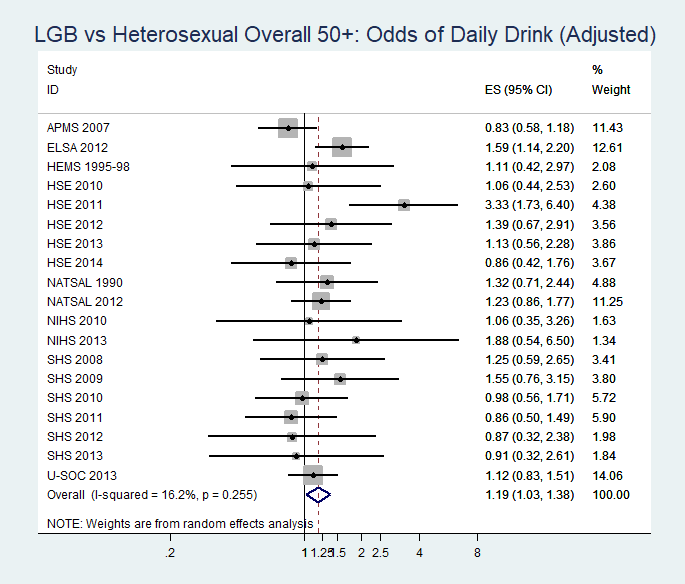


## Males –unadjusted estimates with totals in brackets


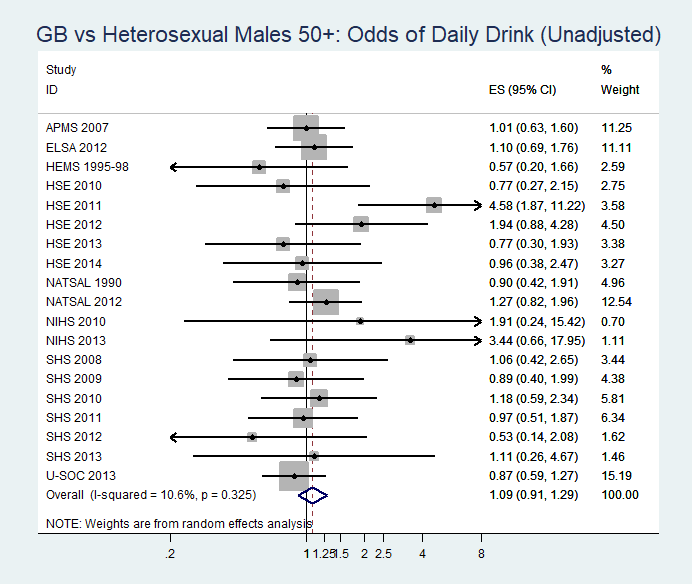


## Males –adjusted estimates with totals in brackets


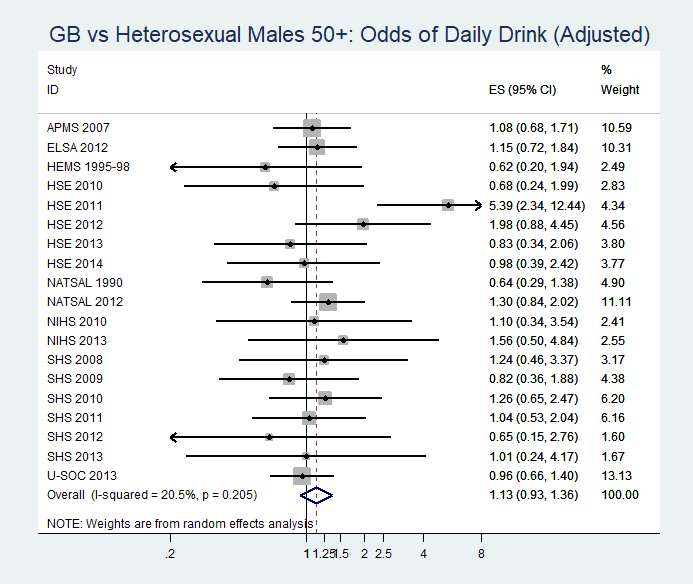


## Females –unadjusted estimates with totals in brackets


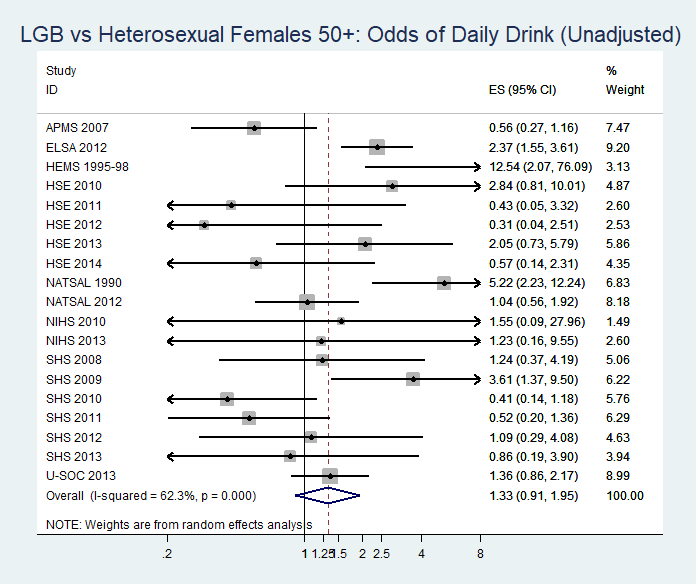


## Females –adjusted estimates with totals in brackets


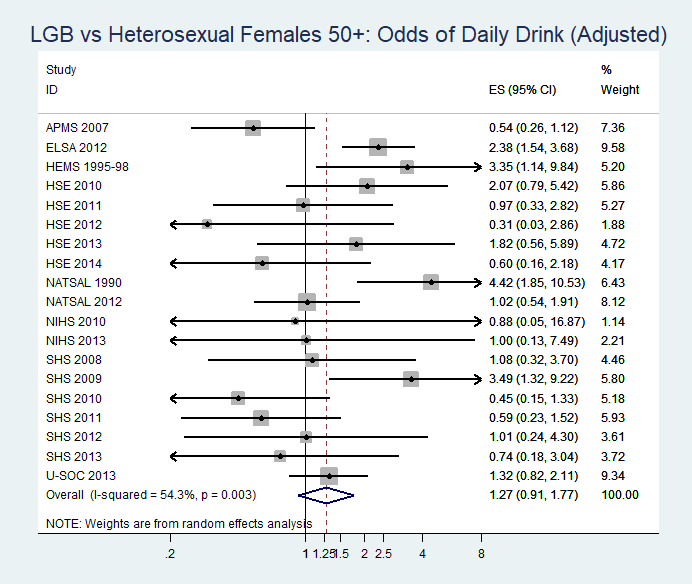


## Further data notes

| Data source abbreviation | Question Details | Model details |
| --- | --- | --- |
| APMS 2007 | Threshold: Drinks alcohol 4 times a week or more |  |
| ELSA 2012 | How often respondent has had an alcoholic drink during the last 12 months  Threshold: 5/6 times a week or almost every day |  |
| HEMS 1995-98 | Consumption of 22 units per week or more | Approximate Bayesian Logistic models used for adjusted estimates for female model |
| HSE 2010 | Threshold: 5/6 times a week or almost every day | Approximate Bayesian Logistic models used for adjusted estimates for female model |
| HSE 2011 | Threshold: 5/6 times a week or almost every day | Approximate Bayesian Logistic models used for adjusted estimates for female model |
| HSE 2012 | Threshold: 5/6 times a week or almost every day |  |
| HSE 2013 | Threshold: 5/6 times a week or almost every day |  |
| HSE 2014 | Threshold: 5/6 times a week or almost every day |  |
| NATSAL 1990 | Average frequency of alcohol consumption  Threshold: everyday or nearly everyday |  |
| NATSAL 2012 | Drink 5+ days per week |  |
| NIHS 2010 | How often respondent has had an alcoholic drink during the last 12 months  Threshold: 5/6 times a week or almost every day | Approximate Bayesian Logistic models used for adjusted estimates for female, male and combined models |
| NIHS 2013 | ow often respondent has had an alcoholic drink during the last 12 months  Threshold: 5/6 times a week or almost every day | Approximate Bayesian Logistic models used for adjusted estimates for male model |
| SHS 2008 | Drinking more than weekly limits |  |
| SHS 2009 | Drinking more than weekly limits |  |
| SHS 2010 | Drinking more than weekly limits |  |
| SHS 2011 | Drinking more than weekly limits |  |
| SHS 2012 | Drinking more than weekly limits |  |
| SHS 2013 | Drinking more than weekly limits |  |
| U-SOC 2013 | How often respondent has had an alcoholic drink during the last 12 months  Threshold: 5/6 times a week or almost every day |  |

# Appendix 10 – Forest Plots and further details for Current Smoking

Please note that the citation for IHS 2014 data is: Office for National Statistics. Social Surveys Division, *Integrated Household Survey 2009-2014: Secure Access* [computer file]. Colchester, Essex: UK Data Archive [distributor], November 2016. SN 8075, http://dx.doi.org/10.5255/UKDA-SN-8075-1

## Overall – unadjusted estimates with totals in brackets


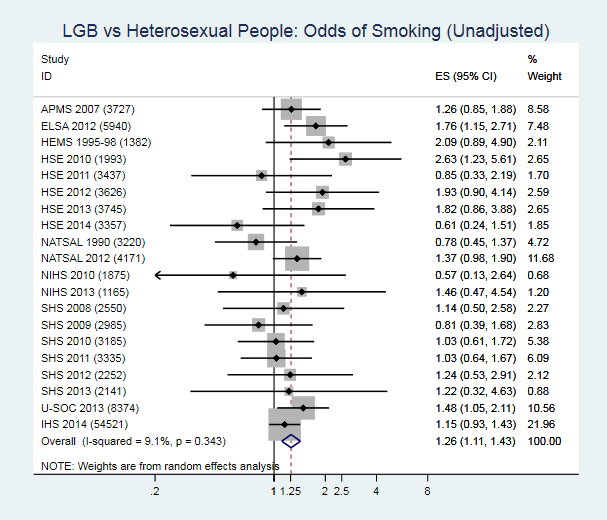


## Overall –adjusted estimates with totals in brackets


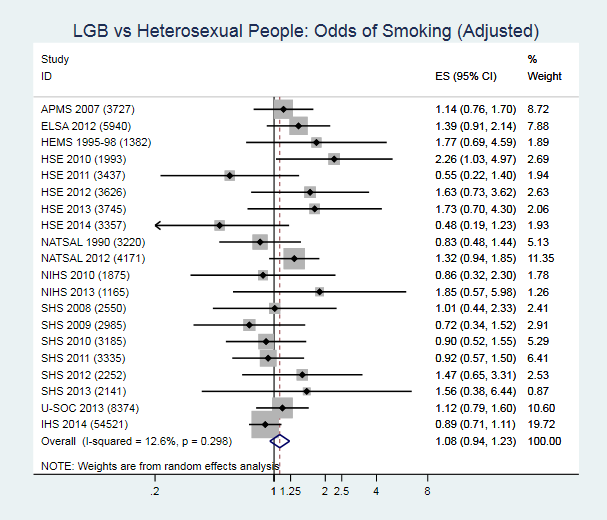


## Males –unadjusted estimates with totals in brackets


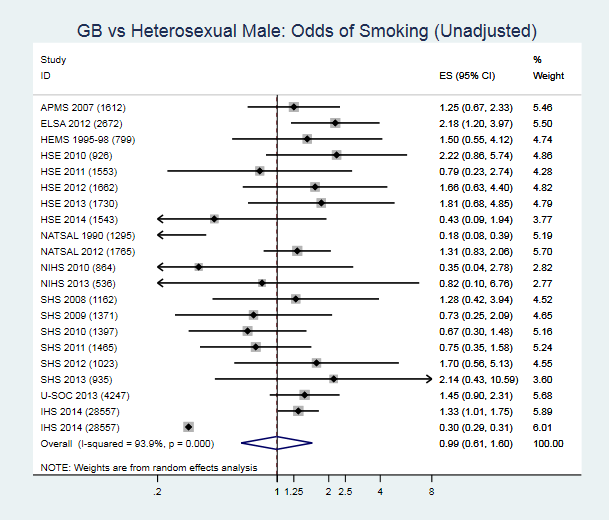


## Males –adjusted estimates with totals in brackets


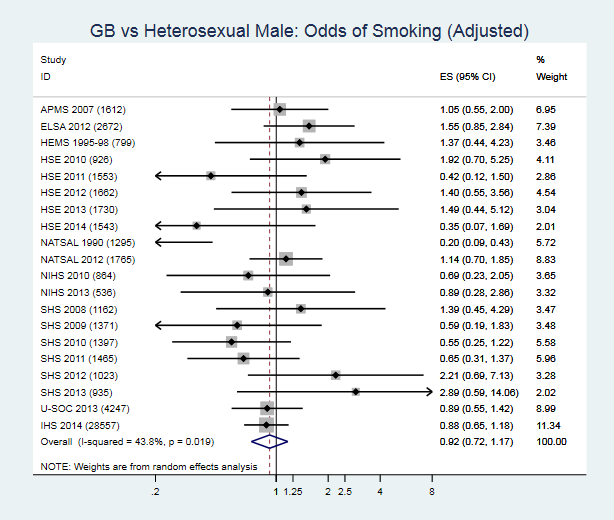


## Females –unadjusted estimates with totals in brackets


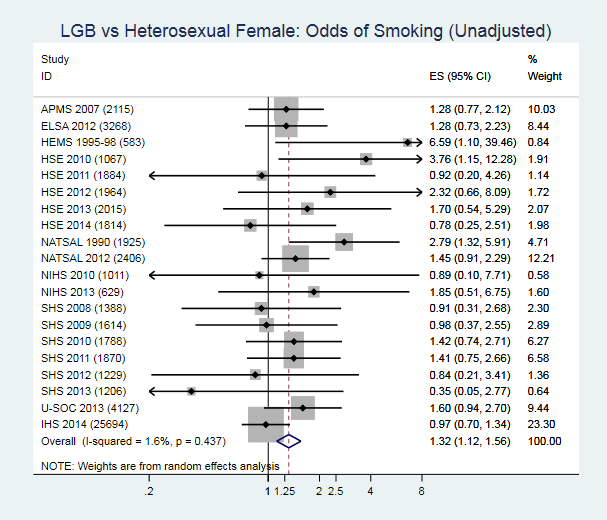


## Females –adjusted estimates with totals in brackets


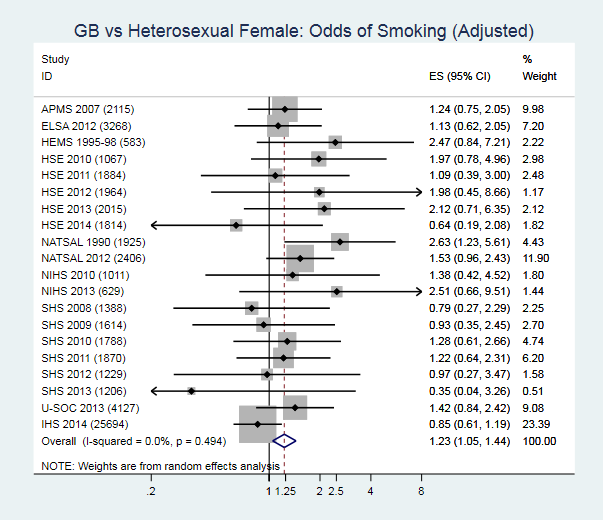


## Further data notes

| Data source abbreviation | Question Details | Model details |
| --- | --- | --- |
| APMS 2007 | Current smoker |  |
| ELSA 2012 | Whether smokes cigarettes at all nowadays? (Threshold: Yes) |  |
| HEMS 1995-98 | Smokes at least one cigarette per day | Approximate Bayesian Logistic models used for adjusted estimates for female model |
| HSE 2010 | Current cigarette smoker | Approximate Bayesian Logistic models used for adjusted estimates for female model |
| HSE 2011 | Current cigarette smoker | Approximate Bayesian Logistic models used for adjusted estimates for female model |
| HSE 2012 | Current cigarette smoker |  |
| HSE 2013 | Current cigarette smoker |  |
| HSE 2014 | Current cigarette smoker |  |
| NATSAL 1990 | Current cigarette smoker |  |
| NATSAL 2012 | Current smoker |  |
| NIHS 2010 | Whether smokes cigarettes at all nowadays? (Threshold: Yes) | Approximate Bayesian Logistic models used for adjusted estimates for female, male and combined models |
| NIHS 2013 | Whether smokes cigarettes at all nowadays? (Threshold: Yes) | Approximate Bayesian Logistic models used for adjusted estimates for male model |
| SHS 2008 | Current cigarette smoker |  |
| SHS 2009 | Current cigarette smoker |  |
| SHS 2010 | Current cigarette smoker |  |
| SHS 2011 | Current cigarette smoker |  |
| SHS 2012 | Current cigarette smoker |  |
| SHS 2013 | Current cigarette smoker |  |
| U-SOC 2013 | Whether respondent reports currently smoking |  |
| IHS 2014 | Whether a current cigarette smoker |  |
